# Nutrition and Supplementation in Soccer

**DOI:** 10.3390/sports5020028

**Published:** 2017-05-12

**Authors:** César Chaves Oliveira, Diogo Ferreira, Carlos Caetano, Diana Granja, Ricardo Pinto, Bruno Mendes, Mónica Sousa

**Affiliations:** 1 Instituto Politécnico de Viana do Castelo - Escola Superior de Desporto e Lazer, Viana do Castelo 4960-320, Portugal; 2 Benfica LAB, Sport Lisboa e Benfica, Lisbon 1500-313, Portugal; dferreira@slbenfica.pt (D.F.); ccaetano@slbenfica.pt (C.C.); dgranja@slbenfica.pt (D.G.); rpinto@slbenfica.pt (R.P.); bmendes@slbenfica.pt (B.M.); 3 Instituto Politécnico de Leiria - Escola Superior de Saúde, Leiria 2411-901, Portugal; monica.sousa@ipleiria.pt

**Keywords:** soccer, football, nutrition, supplementation, recovery, periodization, exercise, food, match

## Abstract

Contemporary elite soccer features increased physical demands during match-play, as well as a larger number of matches per season. Now more than ever, aspects related to performance optimization are highly regarded by both players and soccer coaches. Here, nutrition takes a special role as most elite teams try to provide an adequate diet to guarantee maximum performance while ensuring a faster recovery from matches and training exertions. It is currently known that manipulation and periodization of macronutrients, as well as sound hydration practices, have the potential to interfere with training adaptation and recovery. A careful monitoring of micronutrient status is also relevant to prevent undue fatigue and immune impairment secondary to a deficiency status. Furthermore, the sensible use of evidence-based dietary supplements may also play a role in soccer performance optimization. In this sense, several nutritional recommendations have been issued. This detailed and comprehensive review addresses the most relevant and up-to-date nutritional recommendations for elite soccer players, covering from macro and micronutrients to hydration and selected supplements in different contexts (daily requirements, pre, peri and post training/match and competition).

## 1. Introduction

Association Football, more commonly known as “football” or “soccer”, is the world’s most popular sport [1]. The physical demands of elite match-play have been increasing within the past few decades [2,3]. The modern match also includes more passes, runs with the ball, dribbles and crosses, which collectively suggest a significant increase in the “tempo” of matches [4,5]. The number of matches per season has also increased, with elite clubs frequently playing over 60 competitive matches over a season [4]. Periods of fixture congestion (i.e., 1–3 matches per week) are common in elite soccer [6] and may be further complicated by travel issues during European/World competitions and/or national team fixtures, leading to increased fatigue of soccer players. This, combined with an inadequate recovery, can potentially lead to underperformance and**/**or an increased risk of injury [7,8]. 

Achieving the highest performance during training and competition, improving and accelerating recovery, achieving and maintaining an optimal body weight and physical condition, and minimizing the risk of injury and illness are key issues in contemporary elite soccer. Different fields of scientific knowledge have addressed all these issues, including the field of nutrition [9,10,11,12,13], where specific recommendations have been developed for soccer players. As the physiological demands of soccer are challenging and vary greatly depending on the nature of training, playing schedules and intensity of play, sound dietary practices should be followed [13,14]. This review gathers the most relevant and up-to-date nutritional recommendations for elite male soccer, covering from macro and micronutrients to hydration and selected supplements. Here, readers can find detailed information on the appropriate intakes for each of these nutrients, in different contexts (daily requirements, pre, peri and post training/match and competition).

## 2. Methods

For this review, databases PubMed and Scopus were used and searches were performed up to December 2016. Combinations of the following keywords were used as search terms: “nutrition”, “soccer”, “football”, “team sports”, “body composition”, “carbohydrates”, “fat”, “protein”, “micronutrients”, “iron”, “vitamin D”, “antioxidants”, “hydration”, “supplements”, “creatine”, “beta-alanine”, “caffeine”, “nitrate”, “sodium bicarbonate”. Manuscripts were individually selected for their relevance but no specific scientific approach was used in their selection. References of retrieved articles were used whenever they were considered relevant. Additionally, the books Soccer Science [15], Clinical Sports Nutrition [16] and Science and Soccer: developing elite performance [17] were used as complements.

## 3. Exercise Physiology in Soccer

Soccer is an intermittent team-based sport. Here, elite players perform low-intensity movements (e.g., walking, jogging, standing) for more than 70% of the match, interspersed with approximately 150–250 intense actions that include maximal sprinting, turning, tackling and jumping, as well as accelerations and decelerations [18,19,20]. During a soccer match, fatigue may occur temporarily after short, intense periods during both halves and progressively towards the end of each half [21]. The total distance and high-intensity activities have been found to decrease following the most demanding 5-min periods during a match and at the end of the second half compared with the first half [20]. Match analysis also suggests that the distance covered in high-intensity running by elite players in the last 15 min of a match is 14–45% lower than that observed during the first 15 min [20]. However, the distance run at high intensities can remain constant throughout the second half, due to pacing strategies [22], whereby players perform fewer actions at low or moderate intensities to spare their efforts [23]. Finally, jumping, sprinting and intermittent exercise performance, when evaluated after a match, seems to be significantly lower, compared to baseline values [24,25,26]. Collectively, these findings suggest that, at an elite level of play, players experience fatigue towards the end of the match and temporarily following intense bursts. Regarding positional differences, a significantly greater total distance covered during elite soccer match play has been shown in central midfielders and wide midfielders (both about 12 to 13 km), whereas central defenders have been consistently shown to complete the least total distance (about 10 km or less). Accordingly, central defenders also cover the shortest distance at high intensity and combined high-intensity running and sprinting [27,28]. Regarding sprint activities, wide midfielders and attackers are those who cover the greatest distance during match-play [2]. Mean recovery time between high intensity actions has also shown positional differences, with central defenders generally having more time to recover than others, as opposed to wide defenders [29]. In summary, soccer is characterized by the execution of anaerobic actions that are performed against a backdrop of aerobic energy supply. Nevertheless, and although high-intensity actions make up for a relatively low percentage of the match, these actions cannot be underestimated as they can reveal critical to the outcome of a competition [30].

## 4. Body Composition in Soccer

Athletes who are involved in team sports such as soccer, covering significant distances during a match, are generally aided by a lighter and leaner physique [31,32]. A lean body, with a greater muscle-to-fat ratio, is often advantageous in sports where speed is involved [33], as the storage component of body fat may act as a dead weight to be lifted against gravity during jumping and sprinting (e.g.,). In turn, this affects energy expenditure [34] and is inversely related to aerobic capacity, power-to-weight ratio, and thermoregulation [35]. Nevertheless, the body fat levels of team sport players are not as low as those typically found in endurance athletes such as runners and cyclists [32]. 

Some investigations explored the association between body composition (namely body fat or adiposity) and physical performance in soccer [36,37,38,39,40,41,42,43]. Brocherie and collaborators [41] found that the sum of six skinfolds associated with adipose mass index was largely correlated with speed decrements in a repeated-sprint ability (RSA) test in a group of 16 players from senior male Qatar national team. Furthermore, Silvestre and collaborators [44] showed that reduced body fat levels were associated with improved sprint performance and jump height in a sample of 27 male collegiate soccer players at the beginning of the season. When discussing their research in power testing, Nikolaidis and collaborators [42] found a positive correlation between body fat with mean and 20-m sprint times of a RSA test, in 36 male semi-professional Greek soccer players. More recently, a similar trend was seen [43] where the body fat level of 181 adult soccer players from third and fourth Greek national divisions positively correlated to 20-m sprint times. Together, these data seem to support an association between body fat levels and sprint performance (i.e., lower body fat associated with better sprint performance).

Currently, there are no defined optimal overall body composition values for soccer players [45], although several investigations have reported on the body composition of professional adult male players [45,46,47,48,49,50,51,52,53,54]. Overall, these studies showed that the percentage of body fat ranged from 6% to 20% and that for elite soccer players from the English Premier League—one of the most representative/competitive soccer leagues—values of 10.6% [52], 11.2% [45] and 10.0% [54] were found. Normative data regarding the sum of eight skinfolds through the standardized ISAK protocol is scarce. Hencken and White [49] evaluated the sum of skinfolds of 24 professional soccer players from an English Premier League club and reported a range between 57.9 and 62.5 mm. Positional differences were reported in some studies, with the main difference being goalkeepers evidencing higher body fat values compared to field players [47,53,55,56]. However, this is not always the case [54,57]. Very limited information is available on seasonal differences in body composition changes among playing positions [52] but in a recent study [57] the players of a professional soccer team were evaluated at multiple times across the season and the changes found were similar for all playing positions. The authors concluded that players undergo changes in their fat mass, fat-free soft tissue mass and mineral mass across the season, irrespective of the playing position. Seasonal trends reflect an increase in body fat levels during the offseason, which are then reduced during the preseason, when training volume is highest [53,57]. Another study also found that body composition of elite soccer players changed throughout the competitive season. Specifically, fat mass decreased from the start of preseason to the start of the competitive season. Nevertheless, by the end of the competitive season, it had returned to start of preseason values. Fat-free mass significantly increased from start of preseason until the start of the season and these changes were maintained over the entire competitive season [58]. Age, sex, genetics, and the requirements of the sport are factors that impact the individual athlete’s body composition [33]. Thus, it may be useful for practitioners to provide individualized target zones for players. These targets must then be prospectively fine-tuned according to feedback of a player’s performance, health and well-being.

Techniques commonly used to evaluate an athlete’s body composition include dual energy X-ray absorptiometry (DXA), air displacement plethysmography, hydrodensitometry, skinfold measurements and single and multi-frequency bioelectrical impedance analysis (BIA) [12]. For a detailed comparison between each of these methods, the reader is referred to the review by Ackland et al. (2012) [59].

## 5. Energy Requirements in Soccer

For an elite soccer player, it is very important to provide adequate energy to meet the challenges of high-intensity, intermittent exercise. Several studies have estimated and measured total energy expenditure in soccer players using doubly labeled water, heart rate, video match analysis and activity record monitoring [60,61,62]. Mean energy expenditure (above rest) for a match has been estimated to be approximately 1107 Kcal [62], whereas 3442 to 3824 kcal per day were estimated for daily training [60]. More recently, Anderson et al (2017) reported that mean energy expenditure of elite soccer players was approximately 3,566 kcal during a seven-day period including five training days and two matches [63]. However, besides the impact of individual factors (i.e., body size and composition), there are large differences in energy expenditure depending on training load, player position, environmental conditions and tactics [6,13,64]. The use of heart rate monitors and GPS systems might be useful as they can provide some estimation of individual energy expenditures (based on algorithms) during training, but accuracy can vary greatly among systems, and values should be interpreted with caution [13].

To date, to the authors’ knowledge, only one study directly assessed whether elite adult soccer players could maintain energy balance throughout training and competition [63]. In this investigation, energy intake measured through analysis of dietary records and 24 h recall (~3186 kcal) was not statistically different from energy expenditure measured by doubly labeled water method (~3566 kcal) [63]. In another study, from Bettonviel et al. (2016), although mean energy intake (~2988 kcal) was lower, the lack of changes in body weight during the study period also suggests that players were able to maintain energy balance [65]. These two investigations also showed evidence of energy intake periodization, that is, players adjusted their intake according to the different training and match demands [63,65].

Players must therefore balance overall energy intake per training and competition demands as well as individual body composition goals, while reaching key macronutrient targets, as discussed below.

## 6. Nutritional Strategies to Promote Optimal Performance

In this section, we review the most up-to-date nutritional strategies to promote optimal performance in soccer, covering macronutrients and hydration, micronutrients and supplementation.

### 6.1. Carbohydrates

Carbohydrates (CHO) are considered of vital importance in sports in general and in soccer in particular, as muscle glycogen is the predominant substrate for energy production during a match. After this type of effort, nearly half of vastus lateralis muscle fibers have been classified as empty or near empty in relation to their glycogen content [26]. As such, glycogen depletion is commonly cited as a contributing factor for the progressive fatigue observed towards the end of a match [6,66]. Therefore, athletes should adopt specific nutritional strategies to maximize muscle glycogen content and exercise performance at critical moments such as match play, by manipulation of CHO daily needs and before, during and after exercise/match play. The following position is summarized in Table 1.

#### 6.1.1. Daily Requirements

General nutritional recommendations for peak performance promote strategies to achieve optimal muscle glycogen concentrations through means of high CHO availability. In sports where physical activity duration ranges between 1 h and 2 h, it is believed that athletes should consume 5 to 10 g/kg/day [12]. However, recent research has provided new insights into the interactions of exercise with low CHO availability, whereby the acute and chronic adaptive responses to training or recovery are enhanced in an environment of low exogenous and endogenous CHO stores, since consistently high levels of muscle glycogen seem to attenuate training adaptations [75]. Therefore, researchers are now exploring if a low CHO availability at less relevant moments (such as some training sessions) can potentiate the exercise effects, while maintaining high CHO availability at critical moments (match-play), thereby improving soccer athletes’ performance. 

Anderson and colleagues [6] quantified the daily training and accumulative weekly load (reflective of both training and match play) in professional soccer players during a one, two and three match per week schedule and found evidences of training periodization. Specifically, results showed that training load was progressively reduced in the three days prior to match day (one match per week); that daily training load and periodization was similar in a one and two match per week schedule (although total accumulative distance was higher in a two-match week); and that daily training total distance was lower in a three-match week (although accumulative weekly distance was highest and more time was spent in high speed zones). As such, these authors suggested that CHO intake should also be periodized according to training periodization, suggesting high CHO availability on the day before match, on the day of the match and on the day after match on both one match and two match per week schedule, and a reduced CHO availability on the other days. Given the extreme frequency of match play on a three match per week schedule, these researchers do not advise adopting a low CHO availability in these conditions.

CHO periodization has already been implemented to assess its effects on athletes’ performance, although in other sports than soccer. One practical example cycle consists of (1) late-afternoon scheduling of a high-intensity training session undertaken with high glycogen stores; (2) withholding the ingestion of CHO after the session to maintain glycogen depletion during the overnight recovery period; and (3) at low–moderate intensity steady-state exercise session the following morning completed after an overnight fast [76], or in other words, “train high, sleep low, train low”, or more simply, “sleep low”. Marquet and colleagues [77] performed a three-week training-diet intervention comparing the effects of the “sleep low” method to a standard high CHO availability every day (6 g CHO/kg) and found that the short-term periodization of dietary CHO availability around selected training sessions promoted significant improvements in submaximal cycling economy, as well as supramaximal cycling capacity and 10-km running time in trained endurance athletes. The authors proceeded to see if a shorter exposure to this CHO periodization strategy would be successful in inducing metabolic adaptations and performance improvement and concluded that implementing the “sleep-low” strategy for one week improved performance by the same magnitude previously seen in a three-week intervention, without any significant changes in selected markers of metabolism [76]. The effects of CHO periodization in soccer are still unknown but one can expect future developments on the subject, to detect if and which type of periodization can be most successful for soccer athletes’ performance.

#### 6.1.2. Pre-Exercise/Match

Given the relevance of muscle carbohydrate content for exercise performance, every feeding opportunity may matter for achieving the highest values. A pre-exercise/match diet rich in CHO augments muscle glycogen content as previously observed on a 90 min (four-a-side) soccer play study [69]. Here, six male football players competed on two occasions following an exercise and diet with either high or low CHO regimen (65% vs. 30% of total daily energy intake), designed to manipulate muscle glycogen concentrations. Researchers found that pre-match muscle glycogen concentrations following the high CHO diet were significantly higher than following the low CHO diet. Most importantly, players performed significantly more high-intensity exercise in the match played following the high CHO diet, without any observed differences on the evaluated technical variables.

Regarding the pre-event meal, a study [78] evaluated the effects of ingesting a CHO meal (2.5 g CHO/kg) after an overnight fast. Vastus lateralis muscle biopsies were obtained before and 3 h after the meal and revealed that the CHO meal resulted in an approximate 10% increase in muscle glycogen concentration [78]. Furthermore, it was shown that the intake of a substantial amount of CHO (~200–300g) in the 2–4 h before exercise could prolong endurance performance [79,80,81]. As such, the ingestion of 1–4 g/kg of CHO in the last 4h before match may be recommended, depending on players’ tolerance and individual preferences. The last meal should ideally take place 3–4 h before match and include easy-to-digest foods. The meals 4 h before the start of competition (main meals, e.g., lunch) can contain more carbohydrates, with smaller meals (intermediate meals, e.g., afternoon snack) being targeted for occasions when less time is available prior to the start of competition. Within 60 min before the match, usually until warm-up, light snacks containing high CHO (25–30 g) may further increase the availability of CHO before match, thus sparing liver glycogen. However, there are still some concerns about reactive hypoglycemia (due to increases in insulin production) when CHO are consumed within the last hour prior to exercise [82]. Although it is reasonable to consider that higher pre-exercise insulin concentrations may affect exercise performance by glycemic disposal at the start of the exercise, studies have shown that this does not seem to compromise exercise performance [83]. To this respect, we believe that the precise timing and quantity of feeding within 1 h prior to match should be based on individual preference. It is also important to ensure that the pre-event meal is composed of familiar foods to avoid gastrointestinal issues that many athletes experience before big events. Nervousness and unfamiliar food can sometimes lead to stomach cramps, nausea and diarrhea. Keeping meal choices simple and familiar may be the most important concept for successful pre-event fuelling.

Another strategy used in endurance sports to maximize muscle glucose content before an exercise event is glycogen supercompensation. This can be interesting in isolated events, occurring from time to time, but its effects are less known in intermittent high-intensity sports like soccer, where maximum performance is needed frequently, often more than once a week. McInerney and colleagues [84] tested six trained athletes on three exercise trials, with each exercise bout separated by 48 h. Twenty-four hours before day one, subjects consumed a moderate (6 g/kg) CHO diet, followed by five days of a high (12 g/kg) CHO diet. After each exercise session, subjects were fed a high-CHO meal and monitored during the subsequent 3 h of recovery. Before and immediately upon completion of each of these three exercise bouts, a muscle biopsy was taken. As a result of this protocol, researchers found that well-trained men cannot repeatedly supercompensate muscle glycogen content after a glycogen-depleting exercise and two days of a high-CHO diet, suggesting that the mechanisms responsible for glycogen accumulation are attenuated as a consequence of successive days of glycogen-depleting exercise. Furthermore, exercise performance was similar on days three and five despite the lack of glycogen accumulation on day five. Therefore, the intake of extremely high doses of CHO (i.e., 12 g/kg) may not be a useful nutritional strategy for soccer players. The intake of 6–10 g CHO/kg is generally recommended during the 24 h period before a match [6].

#### 6.1.3. During Exercise/Match

The benefits of ingesting CHO during endurance exercise are well established, and general recommendations for sports where exercise duration ranges from 1 to 2.5 h advocate an ingestion of 30–60 g/h [12]. It is known that athletes’ performance tends to drop over the course of a soccer match [85] and thus, an adequate CHO supply during exercise may attenuate the progression of players’ fatigue. In fact, CHO intake at a rate of 30–60 g/h has been associated with a consistent beneficial effect on performance in soccer [73]. Nicholas and colleagues [86] undertook a study in which they provided soccer players with either a 6.5% CHO solution or a taste- and color-matched placebo solution between the 15-min activity blocks of the Loughborough Intermittent Shuttle Running Test (LIST—designed to simulate the activity pattern characteristic of soccer and other stop-start sports). After performing 75 min of the LIST, players performed alternated 20-m sprints with jogging recoveries to fatigue. Researchers found that intake of the CHO solution allowed athletes a 33% greater running time, than when they ingested the placebo [87]. More recently, others [86] found that when adjusting for body mass, ingesting a 7% CHO intake (49 g) was associated with improved time to fatigue during a simulated soccer match.

Overall performance in soccer is not only dependent on physical power. Motor skills and cognitive performance also play a crucial role on the performance of soccer players and there is a tendency for players’ skills and cognitive performance to decline during the latter stages of a match. CHO has been shown to attenuate or even eliminate this detrimental effect over the course of a match [88,89,90]. As Russell and Kingsley [70] recently reported in a systematic review, six out of eight studies found that the ingestion of 30–60 g CHO/h (via a 6–8% solution of glucose, sucrose, or maltodextrin) was associated with an enhancement of at least one aspect of soccer skill performance.

Adding a small amount of protein to a CHO supplement may further enhance performance as found by Alghannam [91]. In this case, a beverage containing 4.8% CHO plus 2.1% protein ingested prior to and during a 75-min football-specific intermittent exercise, allowed for a post-exercise longer run time to fatigue and lower ratings of perceived exertion than an isolated 6.9% CHO supplement.

Finally, a simple carbohydrate mouth rinse has also shown to be able to produce similar effects, with improvements typically between 2% and 3% during exercise lasting approximately 1 h [92,93]. The mechanisms associated with this phenomenon are yet to be perfectly understood but it is already known that they are specific to carbohydrates and independent of taste [94]. These results suggest that it may not be necessary to ingest large amounts of carbohydrate when an athlete is going to exercise/play for approximately 30 min to 1 h. It is also noteworthy to mention that most studies have been conducted on a cycle ergometer and that some did not produce beneficial results [95]. Nevertheless, an actual CHO ingestion is recommended in most situations, except when an eventual gastrointestinal discomfort is suspected to outweigh the potential performance benefits.

#### 6.1.4. Post-Exercise/Match

Muscle glycogen depletion induces muscle glycogen resynthesis, even in the absence of post-exercise CHO intake [96]. Nevertheless, ingestion of post-exercise CHO further stimulates muscle and liver glycogen synthesis, up to a 10-fold increased rate, in comparison to a post exercise non-fed state [74]. Considering a typical mean glycogen storage rate of 5–6 mmol/kg wet weight/h, roughly 24 h of recovery may be needed to normalize muscle glycogen levels. During periods of high frequency of competitive matches and/or training, athletes may see their optimal muscle glycogen recovery compromised, which can ultimately lead to lower overall individual performances. Most importantly, and since higher muscle glycogen synthesis rates occur immediately after the exercise bout, special attention must be placed in this early-phase of recovery. Since the interval between training sessions can be shorter than 8 h, CHO intake, in the form of solids or liquids, should start as soon as possible after the first session to maximize the effective recovery time [97]. In these scenarios, recommendations estimate an ingestion of ≥1 g/kg/h [74], during the first few hours after exercise. The type of CHO that is consumed seems to be of lesser relevance, provided it produces a large glycemic and insulinemic response [74], although it should be considered that lower glycemic index CHO generally have higher fiber content and result in higher levels of satiation, so more food has to actively be consumed to match the amount of CHO generally found in higher glycemic index foods. Nevertheless, research has shown that individuals may respond to the glycemic index of foods in many different ways. In one interesting paper, researchers continuously monitored week-long glucose levels in an 800-person cohort, measured responses to 46,898 meals, and found high variability in the response to identical meals [98]. In response to the ingestion of a given food, some participants could see a significant elevation on their glycemic levels, while others saw only a slight increase or even none at all, suggesting that universal dietary recommendations may have limited utility. To optimize recovery, athletes’ reaction to different foods or CHO should be tested in order to select the ones that guarantee a rapid and large glycemic and insulinemic response. Nonetheless, athletes should also select foods (or drinks) based on their personal preference and experience.

When CHO intake is adequate (e.g., >1 g/kg/h), the co-ingestion of protein seems to provide no further effect on glycogen synthesis [99]. Nevertheless, in specific circumstances such as low daily energy intake or when the amount of carbohydrates is insufficient (especially in the first 4 h after exercise), the addition of ≥0.3 g/kg/h of protein to a carbohydrate supplement may accelerate muscle glycogen resynthesis via a synergistic increase in insulin secretion and muscle glucose uptake [74]. In soccer, the combination of glycogen and protein in a post-exercise meal may be especially relevant during pre-season, where weight management is generally regarded as a critical goal. Finally, we recall that a reduced total energy intake (below requirements) may affect CHO kinetics and compromise an adequate muscle glycogen repletion [100].

### 6.2. Proteins

An adequate amount of protein should be ingested daily to guarantee an adequate protein synthesis and recovery [15]. Recently, the Academy of Nutrition and Dietetics, Dietitians of Canada, and the American College of Sports Medicine suggested that athletes’ protein needs range from 1.2 to 2.0 g/kg/day and that higher intakes may be indicated for short periods of intensive training or when energy intake is reduced [12]. In general, soccer players seem to accomplish these guidelines [65,101,102,103]. However, more important than the total amount is the intake profile, which includes characteristics as the amount of protein at each meal, the timing of intake, and the source of protein. 

For young adults, it has been suggested that the dose of 20–25 g is sufficient, and optimal, to maximally stimulate muscle protein synthesis (MPS) after strength exercises [104]. Taking into account the player body size, the protein amount per meal corresponds to 0.25–0.4 g/kg [71,105]. However, a recent study from Macnaughton and collaborators [106] suggested that ingesting 40 g of high-quality protein following a whole body resistance (instead of targeting just the legs) exercise bout was superior in stimulating muscle growth response compared to 20 g. Additionally, for the purpose of enhancement of MPS, the use of intact proteins is advised as opposed to a combination of branch chain amino acids (BCAAs) [71]. Specific studies on soccer players are needed in order to understand which dosage is more appropriated and if specific and different strategies are needed throughout the season. 

Regarding the moment of ingestion, it seems that eating protein immediately after exercise is important when the goal is to stimulate MPS [107]. Moreover, in an elegantly conducted study, Areta et al. [108] demonstrated that MPS is greater when 4 × 20 g of protein is ingested every 3 h after exercise, compared to 2 × 40 g every 6 h or 8 × 10 g each 1.5 h. Therefore, the recommendations are towards the ingestion of 0.4 g/kg/meal, 4 meals/day, instead of the typically skewed pattern of protein intake favoring the evening meal [109] that has been observed in soccer players [102]. Additionally, some studies have shown that ingesting protein before exercise may also be beneficial, probably due to a more rapid availability of amino acids in the acute phase after exercise [110]. Finally, there is evidence that supports the ingestion of 30–40 g casein before sleep for maximal stimulation of MPS at night after a strength exercise session [111,112].

#### 6.2.1. Pre-Exercise/Match

For the purpose of maximal enhancement of MPS, players should ingest 0.25–0.4 g/kg of intact proteins in the meal before exercise [71]. As stated above, special attention should be given to CHO before matches, and protein may not be the primary nutritional concern. Nevertheless, protein ingestion before exercise allows post-exercise MPS rates to be elevated more rapidly during the early stages of recovery due to a greater amino acid availability [113]. Since a soccer match leads to extended muscle damage [114], pre-match protein ingestion should also be a priority, especially on a multiple match per week schedule.

#### 6.2.2. During Exercise/Match

Although there are some studies [115,116] showing positive effects of ingesting protein during exercise, its benefits are not yet consensual [113,117]. Given the duration of a typical exercise training and match (<3 h), football might not be the modality that most benefits from protein ingestion during exercise [113]. 

#### 6.2.3. After Exercise/Match

In order to enhance protein synthesis and to allow repair, remodeling and adaptation, players should ingest 0.25–0.4 g/kg in the early recovery period [71,105,118]. Moreover, there is evidence that supports ingestion of 30–40 g casein before sleep for maximal stimulation of MPS at night after a strength exercise session [111,112].

### 6.3. Fats

Fat is a source of energy, fat-soluble vitamins (A, D, E, K), and essential fatty acids. Although the benefits of fat for exercise performance are equivocal [119], fat intake is essential for health and a diet too low on fat has the potential to compromise it, as it reduces the absorption of fat-soluble vitamins and glycogen storage in the muscle [12,13]. The amount of required fat depends largely on the training status and the athlete’s goals [120,121]. Three of the most accredited associations related to sports nutrition (American College of Sports Nutrition (ACSN), International Olympic Committee (IOC) and International Society for Sports Nutrition (ISSN)) [120] recommend a daily fat intake for athletes between 20% and 35% of total energy intake, adding that fat intake should not decrease below 15%–20%. It is suggested that athletes should be cautious of high-fat diets (>30% of total energy intake), as favoring this nutrient can be at the expense of a lower CHO intake and have negative effects on training performance [120].

Alongside with the amount, the type of dietary fat intake should also be considered. Polyunsaturated n-3 fatty acids are essential for overall health of the athlete [122]. Some evidence suggests that in today’s diet, the ratio of n-6 to n-3 fatty acids ranges from 10:1 to 20:1 [13,122], which can result in excessive inflammation and detrimental post-exercise recovery. Therefore, a regular supply of foods rich in n-3 should be part of the daily menu plan [13] to increase the production of endogenous antioxidant enzymes [122] and increase oxygen delivery to the heart muscle [123]. Finally, fish oil concentrates rich in eicosapentaenoic acid (EPA) and docosahexaenoic acid (DHA) have been used to counteract the effects of the inflammatory state [124] and dietary fish oil supplementation has a marked protective effect in suppressing exercise-induced bronchoconstriction in elite athletes [122].

#### 6.3.1. Pre-Exercise/Match

Fat is generally recommended to remain at low levels in the pre-exercise meals, to avoid interfering with gastric emptying or causing gastrointestinal problems [12]. 

#### 6.3.2. During Exercise/Match

The use of medium chain triglycerides (MCTs) has been hypothesized to benefit sport performance due to their enhanced digestibility and metabolization when compared to other fats [125]; however, it is unlikely that the provision of MCTs during exercise can promote a sufficiently large increase in rates of fat oxidation to support glycogen sparing and performance benefits, and there is a substantial risk that gastrointestinal disturbances may actually impair performance [16]. Therefore, the consumption of fats during training sessions and matches is not advised for soccer players. 

#### 6.3.3. After Exercise/Match

The minimal available evidence suggests that the ingestion of low to moderate amounts of fat do not impair glycogen [126,127] nor muscle protein resynthesis [128], if adequate amounts of carbohydrates and protein are consumed. Excessive fat may, however, displace carbohydrate foods within the athlete’s energy requirements and gastric comfort, thereby indirectly interfering with glycogen storage.

### 6.4. Hydration

During sports events, when the internal body temperature rises, the main mechanism contributing for increasing heat losses is the activation of sweat glands. Indeed, the evaporation of water through sweat on the skin surface is a very efficient mechanism for removing heat from the body [129,130]. Losses through sweat vary greatly between individuals and depend upon environmental conditions [131,132] as well as other individual characteristics such as body weight [133], genetic predisposition, heat acclimatization state [134], and metabolic efficiency (economy at undertaking a specific exercise task). In a soccer match, sweat rates will vary between players according to their position and playing style as well as the total time spent on the field [135].

There are several studies that evaluated sweat losses and sweat rates in soccer (see Table 2):

By analyzing Table 2, it is possible to conclude that some percentage of dehydration commonly occurs in soccer players, and a significant amount of fluid can be lost through sweating even when the match is played in a cold weather environment. Equally important but often neglected is the observation that a hypohydrated player at the beginning of a sport event has already a hydration deficit, which can more easily compromise sports performance. A study conducted by Maughan et al (2004) found high levels of urine osmolality in some players, suggesting that they began training sessions already in a hypohydrated state [136]. Opportunities for fluid intake during match period are limited, and the ability to empty ingested fluid from the stomach may be compromised, so it is of great importance for players to ensure they are fully hydrated before beginning either training or match-play [136].

Dehydration predominantly presents a thermoregulatory challenge since skin blood flow is reduced during exercise and plasma volume is depleted due to sweating; this reduces heat dissipation and results in elevated core temperature [140]. Dehydration can develop when body fluid losses exceed fluid intake, and it often occurs during exercise, heat stress, restricted fluid intake, or any combination of these [33,72,141]. It is often described in terms of changes in body mass during acute exercise (2% dehydration is defined as a water deficit equal to 2% of body mass). Usually, only about half of sweat losses are voluntarily replaced during exercise [142,143] which generally results in moderate acute water deficits in team sports (1%–2%) [142]. This information is based on data reported in different soccer studies, shown in Table 2. It is also important to mention that some studies in more extreme conditions such as 27 ± 1 °C; RH = 65 ± 7% [138] and 35 ± 1 °C; RH = 35 ± 4% [132] show dehydration percentages considerably higher (3.4 ± 0.7% and 3.4 ± 1.1%, respectively). Dehydration percentages of 1% or 2% of body water can already have detrimental consequences such as impaired cognitive functions, alertness and physical performance. [130,135,144,145,146]. A dehydration of >2% body mass deficit has been shown to impair football-specific performance, including intermittent high-intensity sprinting and dribbling skills [135,146,147,148] and it is also known that greater levels of dehydration will further degrade aerobic exercise performance [149]. Physiologic factors that contribute to dehydration-mediated aerobic exercise performance decrements include increased body core temperature, increased cardiovascular strain, increased glycogen utilization, altered metabolic function, and altered central nervous system function [150,151].

A good baseline is typically determined via consecutive daily measures of first morning, post void and nude body mass, usually after providing fluid (1–2 L) the evening prior [152,153,154]. If a well-hydrated baseline body mass is not established, it is unclear what degree of dehydration has been achieved by any acute perturbation. Nevertheless, the 2% body mass is a consensus in the literature representing a threshold at which aerobic exercise performance or endurance becomes impaired [72,155], but it is worth noting that exercise-induced dehydration up to 4% of body weight loss may not alter exercise performance, at least while cycling exercise mode [156]. On the other hand, impairments on anaerobic performance and muscular strength remain unclear [155,157]. The signs of mild-to-moderate dehydration include dry, sticky mouth, sleepiness or tiredness, thirst, decreased urine output, muscle weakness, headache, dizziness or light-headedness [130]. It is important to mention that thirst is triggered by increases in plasma and extra cellular fluid osmolarity and by reductions in plasma volume at water deficits that correspond to a body weight loss of 1–3%. This means that, in the rehydration process, thirst can disappear before water balance is reached [130].

Besides water, electrolytes, particularly sodium and chloride in smaller amounts, are also lost with sweat. The other electrolytes present (e.g., potassium, calcium, magnesium) are at vastly lower concentrations [158]. In studies where sweat electrolyte composition is determined, absorbent sweat patches are attached to various anatomical sites after the skin is thoroughly cleaned with deionized water and dried [136]. This procedure helps to identify those players with high sweat sodium losses who may need to pay particular attention to sodium replacement [159]. In soccer there are several studies that report sodium losses from 30 ± 19 mmol/L [159] to 62 ± 13 mmol/L [129], which is equivalent to “salt” losses from 3.9 to 6.1 g. Regarding chloride and potassium, a study of 24 professional male players reported losses of 43 ± 10 mmol/L and 6 ± 1.3 mmol/L, respectively [136]. Although there is a limited number of studies regarding chloride losses in soccer, in what concerns potassium, a study [159] reported slightly different values of 4.2 ± 1 mmol/L. It is clear that high salt losses are a factor in the etiology of muscle cramps and heat illness in industrial settings and that these can be alleviated by the ingestion of salt-containing drinks but it is less clear whether this applies to the generally smaller losses [159]. However, some studies report that people susceptible to muscle cramps are believed to be often profuse sweaters with large sweat sodium losses [160,161,162].

The commonly used technique to measure changes in hydration status is the measurement of body weight changes that occur during short periods of time [163], which is because when an individual is in an energetic balance, a body weight loss essentially equals water loss. Measurements of body weight must be carried out under standard conditions, preferably in the morning in the fasted state and after micturition and defecation [130]. There are, however other options to evaluate changes in hydration status such as the BIA technique [164], although it remains inappropriate for measuring small changes in total body water in the range of 1 L [165,166,167]. Urinary indices of hydration, such as urine osmolarity [168], urine-specific gravity or 24-h urine volume, may also be used, but urine variables often mirror the recent volume of fluid consumed rather than the state of hydration [169]. For example, the intake of a large volume of water rapidly dilutes the plasma and the kidneys excrete diluted urine even if dehydration exists. A number of studies have shown the urine osmolality, measured on samples collected at rest prior to exercise, can be used in athletes as an index of hydration status, being urine osmolality normally less than about 600–900 mosmol/kg in individuals who are well hydrated [136,170]. Even if there is no real consensus with regard to the method by which to measure hydration status, for clinicians and general practitioners, the urine color chart is normally used as an indicator [171]. Finally, an approximation of hydration status can be obtained by measuring the sensation of thirst with a simple numerical scale [130].

In terms of recommendations for adults performing modest physical activity, the daily water needs of men increase from 2–2.5 L (if sedentary) [172] to about 3.2 L, while more active adults living in a warm environment can have daily water needs of about 6 L [173]. Thus, it is certain that physical activity results in increased water requirements [134].

#### 6.4.1. Pre-Exercise/Match

Before exercise, the goal is to start the physical activity euhydrated and with normal plasma electrolyte levels [149]. The prehydration program will help ensure that any previously incurred fluid electrolyte deficit is corrected prior to initiating the exercise task. When hydrating prior to exercise the individual should slowly drink beverages (for example, ~5–7 mL·kg^−1^ per body weight) at least 4 h before the exercise task. If the individual does not produce urine, or the urine is dark or highly concentrated, he should slowly drink more (for example, another ~3–5 mL·kg^−1^) about 2 h before the event. By hydrating several hours prior to exercise there is sufficient time for urine output to return towards normal before starting the event [174]. Another small bolus in the end of warm-up is recommended to replenish sweat losses during this period [174].

Consuming beverages with sodium (20–50 mEq·L^−1^) and/or small amounts of salted snacks or sodium containing foods at meals will help to stimulate thirst and retain the consumed fluids [175,176].

Enhancing palatability of the ingested fluid is one way to help promote fluid consumption, before, during, or after exercise. The preferred water temperature is often between 15 and 21 °C, but this factor, as well as flavor preference varies greatly between individuals and cultures [72]. 

#### 6.4.2. During Exercise/Match 

The amount and rate of fluid replacement depends upon the individual sweating rate, exercise duration, and opportunities to drink. Individuals should periodically drink (as opportunities allow) during exercise whenever possible, if it is expected they will become excessively dehydrated [72]. It is recommended that individuals should monitor body weight changes during training/competition to estimate their sweat lost during a particular exercise task with respect to the weather conditions. This allows customized fluid replacement programs to be developed for each person’s particular needs [72].

Both soccer rules and gastric tolerance do not allow suitable hydration for soccer players [177]. During the match, it is difficult to ingest fluids because there are no breaks for this specific purpose, hence the player’s fluid intake has to be a priority during half-time [136]. Its ingestion should be enough to replace loss from sweat, with volume and contents known by players and based in individual requirements and preferences [178]. These beverages should have a carbohydrate concentration of 6% to 8%, and should be provided at a temperature of 15 to 20 °C at every 15–20 min, in a volume of 150 to 300 mL, to provide substratum, but not to limit rate of gastric emptying [72,136]. 

During training sessions, the need to include carbohydrates and electrolytes will depend on the specific exercise task (e.g., intensity and duration) and weather conditions. The sodium and potassium help to replace sweat electrolyte losses, while sodium also helps to stimulate thirst, and carbohydrate provides energy. These components also can be consumed by non-fluid sources such as gels, energy bars, gums and other foods.

#### 6.4.3. After Exercise/Match

After exercise, the goal is to fully replace any fluid and electrolyte deficit [72]. If players have accrued a body mass deficit, they should aim to completely replace fluid and electrolyte losses prior to the start of the next training session or match. If dehydration is severe (>5% of body mass) or rapid rehydration is needed (e.g., <24 h before next training session or match) the recommendation is to drink ~1.5 L of fluid for each 1 kg of body mass deficit [179]. The additional volume is needed to compensate for the increased urine production accompanying the rapid consumption of large volumes of fluid [176]. Therefore, when possible, fluids should be consumed over time (and with sufficient electrolytes) rather than being ingested in large boluses to maximize fluid retention [180,181]. If recovery time and opportunities permit, consumption of normal meals and snacks with a sufficient volume of plain water and sodium will restore euhydration [149,182].

Dehydration is not the only problem caused by inadequate intake of fluids, because hyperhydration can also occur. Hyperhydration can be achieved by overdrinking combined with an agent that “binds” water within the body, such as glycerol [183,184]. Simple overdrinking will usually stimulate urine production [149,185] and body water will rapidly return to euhydration within hours, [182,186], however, this compensatory mechanism (urine production) is less effective during exercise and there is a risk of dilutional hyponatremia [185].

### 6.5. Micronutrients

Micronutrients (i.e., vitamins and minerals) play an important role in the body because many are precursors for various important physiological processes. Exercise stresses many of the metabolic pathways in which micronutrients are required, and training may result in muscle biochemical adaptations that increase the need for some micronutrients [12].

To ensure sufficient intake, soccer players are encouraged to consume nutrient-dense foods [187]. In special circumstances, such as for players who are following a negative energy balance for weight management purposes or for players who avoid or eliminate large food groups, or consume poorly chosen diets, micronutrient intake may be sub-optimal and thus supplementation may be beneficial [12]. In these situations, the use of a standard multivitamin supplement that is batch tested under the guidance of a qualified sports nutritionist or dietitian may be appropriate to ensure that 100% of the recommended dietary allowance (RDA) for all micronutrients is met [13]. However, as with all nutrients, the focus should first be on nutrient-rich foods, and then a supplement if indicated.

In soccer, special consideration must be given to iron, vitamin D and antioxidants (see below). Vitamins B (B1, B2, niacin, B6, B12, biotin, folic acid and pantothenic acid), which have crucial functions in energy metabolism, tend to be consumed at sufficient quantities in soccer players who meet their increased energy requirements through an adequate and balanced dietary intake [188].

#### 6.5.1. Iron

Iron deficiency, with or without anemia, can impair muscle function and limit work capacity, leading to compromised training adaptation and athletic performance [12]. This is especially important for soccer, due to its heavy reliance in aerobic metabolism [189].

Reinke and colleagues [190] observed a significant proportion (approximately 30%) of absolute (serum ferritin <30 μg/L) and functional (normal ferritin with transferrin saturation <20%) iron deficiency in professional soccer players (*n* = 10) at the end of season. Although holiday period led to increased ferritin levels in these players, this was not sufficient to fully restore an optimal iron status [190]. Other observations [191,192] also support a tendency for iron status disturbances during a soccer season. It was speculated that accumulated fatigue and inadequate recovery time during a competitive in-season period may predispose football athletes to iron status disturbances [190]. Others [193], albeit detecting a significant (approximately 31%) prevalence of iron deficiency/depletion (serum ferritin <30 μg/L) in professional soccer athletes, did not observe significant changes in the iron status during a competitive season. 

Soccer players, especially those at higher risk for deficiency, should aim for an iron intake equal or greater than their RDA (i.e., >8 mg/day for men) [12]. When iron deficiency anemia (IDA) is detected, clinical follow must ensue. For those who have iron deficiency (e.g., low ferritin) without anemia, eating strategies that promote an increased intake of food sources of well-absorbed iron (e.g., heme iron from animal sources, non-heme iron from vegetables plus vitamin C foods) should be promoted as the first line intervention [12]. Oral supplementation with iron tablets (i.e., ferrous sulfate providing 80 mg of elemental iron) may also be useful to correct low ferritin levels [194]. Of note, the intake of iron supplements in the period immediately after strenuous exercise is contraindicated since there is the potential for elevated hepcidin levels to interfere with iron absorption [195].

#### 6.5.2. Vitamin D

It is long recognized that vitamin D regulates calcium and phosphorus absorption and metabolism, and plays a key role in maintaining bone health. Emerging research, however, also highlights the important role of vitamin D for non-skeletal functions including skeletal muscle growth, immune function, inflammatory modulation and athletic performance [196]. 

Soccer players with low levels of 25(OH)D (<30 ng/mL or <75 nmol/L) may be more likely to have musculoskeletal injuries and stress fractures [197]. While it has also been suggested that lower levels of vitamin D may lead to reduced muscle strength [198,199], two studies performed in professional soccer have failed to find a consistent association between vitamin D status and muscle strength variables [200,201]. Several studies assessed vitamin D status in soccer players living in different regions and at different time of the year [200,202,203,204,205]. Vitamin D insufficiency (25(OH)D < 30 ng/mL or 75 nmol/L) was relatively common in players living in countries at higher latitudes (> 35th parallel), especially in winter months [202,203,204]. However, insufficient vitamin D levels were also detected in a large proportion (84%) of players living in Qatar (latitude < 35th) during summer, highlighting the relevance of other factors such as exposure practices, clothing, sunscreen use, skin pigmentation and timing of training sessions (e.g., after sunset) [200].

Although the human requirement can be met entirely through synthesis in the skin upon exposure to sunlight [206], dietary vitamin D may also contribute to promote an adequate status. Vitamin D is found in the diet from foods such as fatty fish and egg yolks but also fortified foods (e.g., milk, yogurt, ready-to-eat cereal), and is well absorbed in association with dietary lipids [196]. The RDA for vitamin D varies according to region, with recommendations ranging from 200 IU in Australia and New Zealand to 600 IU in USA and Canada [207]. Where sensible sun exposure is not possible or desired, athletes with insufficient status require supplementation with at least 1500–2000 IU/day vitamin D to keep blood vitamin D concentration in the sufficient range [208].

Routine screening of vitamin D status may be useful in the athlete [196]. If routine screening is not possible, athletes with low exposure to UVB, history of stress fracture, frequent illness, bone and joint injury, skeletal pain or weakness, or signs of overtraining should be prioritized for assessment [12]. Vitamin D blood levels from 30–32 ng/mL (80 nmol/L) and up to 40 ng/mL (100 nmol/L) to 50 ng/L (125 nmol/mL) have been recognized as prudent goals for optimal training induced adaptation [12].

#### 6.5.3. Antioxidants

Several studies to date have shown inconsistent results indicating either positive or negative effects of antioxidant supplementation combined with training [209,210,211,212,213]. Briefly, the most commonly used arguments to support antioxidant supplementation are: (i) the fact that exercise leads to an increase in ROS production and that increased levels of antioxidants could counteract the ROS, preventing or reducing damage and, therefore, muscle pain [214], (ii) that some antioxidants shown to improve endurance performance [215] and to delay fatigue, and (iii) that some athletes may not achieve the nutritional recommendations for antioxidant intake just with food [216,217,218]. On the other hand, some arguments have been used against antioxidant supplementation, namely: (i) the fact that regular exercise leads to an increase in enzymatic and non-enzymatic antioxidants in muscle fibers [219]; (ii) that antioxidant supplementation may impair muscle function or delay some adaptations induced by exercise [220,221], by interfering with cell signaling functions of ROS, affecting muscular performance [222]; (iii) that antioxidant supplementation does not seem to lead to better outcomes, compared with placebo, regarding muscle function, inflammation [223] and redox status [224] after eccentric exercise; (iv) that antioxidant supplementation may contribute to increase muscle damage and oxidative stress [225], and (v) that some studies do not support the concept that antioxidant supplementation is beneficial to human health [226] and doubts have been placed about the chronic intake effects on performance of antioxidant supplementation in high doses [222,227]. Moreover, it has been reported that the protective effect of a diet, with natural sources of antioxidants, is not equivalent to the protective effect of supplementation [228]. Given these facts, it is currently suggested [209,229] that due to the limited evidence to recommend antioxidant supplements, athletes should rather focus on consuming a well-balanced and energetically adequate diet, which can provide antioxidant-rich foods. For detailed information regarding the impact of natural-present antioxidants on exercise, other review paper is recommended [118]. Nevertheless, new approaches regarding the acutely intake of some antioxidants such as vitamin E and N-acetylcysteine seem promising and are pointing towards an acute performance benefit, but further research is needed [227]. 

### 6.6. Supplementation

It is well known that supplementation is a common practice among soccer players [230]. Despite the large prevalence of use, athletes not always fully understand the possible risks that may arise from dietary supplements (DS) consumption [231,232]. The same supplement may be of use in some circumstances but detrimental to performance in others [233]. Of more concern is the poor quality insurance of DS. Regulation of DS varies between countries, and the increasing market of online sales allows a tremendous offer of different products, sometimes with uncertain origin [233]. In US [234], the country that probably represents the larger DS market, and in Europe [235] DS, independently of their form, are considered to belong to a special category under the general “umbrella” of foods, not drugs. This means that DS are not under the same regulations nor are subject to the strict control that is applied to the pharmaceutical industry. The poor quality of DS is a concern for sports community. There are reports of contamination with impurities [233] and reports of undeclared allergens or microbiological contamination [236]. This scenario may cause acute health consequences and jeopardize a crucial period of training or competition [233]. However, more worrisome is the possible contamination of DS with prohibitive substances in sports [237]. Although there are some products that deliberately contain substances prohibited by the World Anti-Doping Agency [238], others—15% [239] to 25% [240]—are contaminated with them, i.e., these substances are not declared in the label. Some of these cases may result from inadvertent cross-contamination due to poor quality control but others seem to involve deliberate adulteration [236]. Therefore, there is a high and real risk for athletes consuming contaminated supplements to inadvertently fail a doping test [241]. Since the contamination of supplements became a serious issue, reputable supplement companies have been taking several measures to prevent adulteration [238]. These include product and manufacturing audits to attest the quality control along the production process, and the testing of products for trace amounts of prohibitive substances by specialized sports anti-doping laboratories [238].

Recently, the Academy of Nutrition and Dietetics, Dietitians of Canada, and the American College of Sports Medicine [12] divided the DS into three categories: sports foods (such as sports drinks, sports bars and electrolyte supplements), medical supplements (iron, calcium, vitamin D and n-3 supplements) and specific performance supplements (creatine, caffeine, sodium bicarbonate, beta-alanine and nitrate). Considering the specificity of soccer, Table 3 displays the rationale for intake and the protocol and practical recommendations of the specific performance supplements of interest for soccer.

#### 6.6.1. Beta-Alanine

Beta-alanine supplementation has become a common practice among competitive athletes participating in a range of different sports [250]. Among other roles [251], beta-alanine supplementation augments intramuscular carnosine content, leading to an increase in muscle buffering capacity, as carnosine readily accepts protons during contraction-induced acidosis [252]. As such, a delay in the onset of muscular fatigue and a facilitated recovery during repeated bouts of high-intensity exercise have also been reported as positive effects of beta-alanine supplementation [250]. As muscle is unable to synthesize the two carnosine precursors, l-histidine and b-alanine, the concentration of intracellular carnosine is largely dependent on the uptake of these amino acids from the bloodstream. Of the two, beta-alanine has been identified as the rate-limiting precursor to carnosine [253]. Reviews on the effects of its supplementation have been published [242,254].

In a classic study by Hoffman et colleagues [255] college football players ingested 4.5 g of beta-alanine or placebo for 30 days. Beta-alanine supplementation began three weeks before preseason training camp and continued for an additional nine days during camp. Anaerobic performance, training volume, and ratings of soreness and fatigue were assessed pre- and post-intervention and included a 60-s Wingate anaerobic power test and three line drills. At the end of the 30-day investigative period, only the beta-alanine group showed a trend toward lower fatigue rates during the anaerobic performance test. More interestingly for team sports, beta-alanine supplementation allowed for higher training volumes and lower subjective feelings of fatigue, indicated that as duration of supplementation continued, the efficacy of beta-alanine supplementation in highly trained athletes became apparent (training logs were used to record resistance training volumes). Earlier, the effects of creatine and beta-alanine supplementation on performance and endocrine responses in strength/power athletes had been analyzed and it was found that beta-alanine + creatine did not produce further strength increments when compared to creatine alone but the co-supplementation elicited greater changes in lean body mass and percent body fat [256]. 

Studies conducted on the effects of beta-alanine on soccer related performance variables are scarce. Saunders and colleagues gave either a placebo or a beta-alanine supplement (3.2 g/day for 12 weeks) to 17 amateur soccer players and evaluated their effect on the YoYo Intermittent Recovery Test Level 2 [257]. These researchers found that while participants on the placebo group saw a decrease on their performance (−7.3%), participants on the supplemented group achieved the opposite (+34.3%). Also of note, only two participants on the placebo group improved their performance, but eight out of the nine supplemented participants achieved better results, while the remaining participant saw no modification on his performance.

Another interesting study aimed to determine if beta-alanine could help to improve sprinting performance at the end of an endurance competition. This is interesting for team sports, as performance detriments tend to increase by the end of a match and the ability to perform a maximal intensity exercise by then may be decisive for the match result. Therefore, subjects performed a 10-min time trial and a 30-s isokinetic sprint (100 rpm) after a 110-min simulated cycling race and it was found that during the final sprint after the time trial, beta-alanine supplementation resulted on an average increased peak power output by 11.4% and a mean power output increased by 5.0% [258].

A recent position stand of the International Society of Sports Nutrition [243] states that: (1) daily supplementation (4–6 g/day) significantly augments muscle carnosine concentrations; (2) beta-alanine supplementation currently appears to be safe in healthy populations at recommended doses; (3) paresthesia (tingling) may occur as a side effect, but studies indicate this can be attenuated by using divided lower doses (1.6 g) or using a sustained-release formula; (4) daily supplementation (4–6 g/day) for at least two to four weeks has been shown to improve exercise performance, with more pronounced effects in open end-point tasks/time trials lasting 1 to 4 min in duration; (5) beta-alanine attenuates neuromuscular fatigue, particularly in older subjects, and preliminary evidence indicates that beta-alanine may improve tactical performance; (6) combining beta-alanine with other single or multi-ingredient supplements may be advantageous when supplementation of beta-alanine is high enough (4–6 g daily) and long enough (minimum four weeks); (7) more research is needed to determine the effects of beta-alanine on strength, endurance performance beyond 25 min in duration, and other health-related benefits associated with carnosine. In summary, the body of scientific data indicate that athletes may not only be using beta-alanine supplementation to enhance sports performance but also as a training aid to augment bouts of high-intensity training, to decrease rates of perceived fatigue and to perform higher training volumes in team-sport athletes, which may allow for greater overload and superior adaptations compared with training alone [250]. Nonetheless, more studies involving soccer players are needed to fully comprehend the interactions between beta-alanine supplementation and performance in soccer.

#### 6.6.2. Caffeine

The ergogenic effect of caffeine, one of the most popular dietary supplements, is relatively well-documented [259]. The International Society of Sports Nutrition considered that caffeine supplementation is beneficial for intermittent exercise within a period of prolonged duration, including team sports such as soccer [260]. 

Froskett and collaborators [261] showed that the ingestion of 6 mg/kg of caffeine 60 min before a simulated soccer activity could improve players’ passing accuracy and jump performance without any detrimental effects on other performance parameters. In another study [262], male soccer players performed a 90-min intermittent shuttle-running trial 1 h after ingesting a carbohydrate-electrolyte solution containing 3.7 mg/kg of caffeine. The solution was also ingested every 15 min during the exercise protocol. Compared to placebo, the addition of caffeine to the carbohydrate-electrolyte solution improved sprinting performance, countermovement jumping and ratings of pleasure. Based on this two studies, a recent review [70] concluded that caffeine in doses of 6 mg/kg has the potential to preserve skills performed under conditions that induce soccer-specific fatigue, but its effects are not strongly conclusive due to the small number of studies.

More recently, 6 mg/kg of caffeine ingested 1 h before a 90-min intermittent treadmill-running protocol enhanced the ratings of pleasure and arousal during the exercise protocol and increased vigor compared to placebo [244]. In another study [263], also with female players, the ingestion of 3 mg/kg of caffeine in the form of an energy drink 1 h before a countermovement jump and a 7 × 30 m sprint test followed by a simulated soccer match (2 × 40 min) increased the countermovement jump height and the average peak running speed during the sprint test. During the simulated match, the ingestion of caffeine increased the total running distance, the number of sprints and the running distance covered at >18 km/h. Nevertheless, it is worthy to mention that not all studies investigating the use of caffeine on soccer have found positive effects [264,265].

#### 6.6.3. Creatine

Creatine is an endogenous compound synthesized from arginine, glycine and methionine and is mostly (95%) stored in skeletal muscle where it can be found as a free form or bound to a phosphate molecule (phosphocreatine) [245]. The most well know physiology effect of creatine is its role on the maintenance of intracellular levels of adenosine triphosphate (ATP), whereby phosphocreatine promptly donates its phosphate molecule to adenosine diphosphate (ADP) to (re-)form ATP, and therefore, maintaining the highest power output. However, this energy system can be almost completely depleted after only a few seconds of maximal exercise [266]. In a single 6-s sprint, glycogen degradation (glycogenolysis) contributes 50% of the ATP production, whereas phosphocreatine contributes 48% and the remaining 2% is provided by the muscle’s small store of ATP [267]. In even shorter maximal sprint durations, phosphocreatine is the primary energy source for ATP production. Along with power and strength, creatine has traditionally been praised for its effects on muscle mass increase and many other beneficial outcomes, from age-related disease prevention to improved brain performance [245,268], and is a popular supplement among elite soccer players [230].

The effects of acute creatine supplementation have been investigated. Ostojic and colleagues found that creatine supplementation for seven days (3 × 10 g/day) improved performance in a soccer-specific battery of tests, including a dribble test, a sprint-power test, an endurance test, and a vertical jump test [269]. Another study revealed that six days of creatine supplementation (4 × 5 g/day) improved repeated sprint performance and jumping ability after an intermittent exercise test in 17 highly trained male soccer players [270]. The same creatine supplementation protocol produced improved results on repeated sprint and agility tasks in elite female soccer players [271].

Longer creatine supplementation protocols have also been implemented. A 13 weeks of creatine supplementation (2 × 7.5 g/day in the first week and 5 g/day throughout the rest of the protocol) improved the muscle strength of collegiate female soccer players [272]. Claudino and colleagues found that creatine monohydrate supplementation prevented the decrement in lower-limb muscle power in elite soccer players during a 7-week pre-season progressive training [273]. Here, subjects from the creatine group received 20 g/day of creatine monohydrate for 1 week divided into four equal doses (loading phase), followed by single daily doses of 5 g for the next six weeks (maintenance phase). More recently, Ramirez-Campillo and colleagues investigated the effects of a six-week plyometric training and creatine supplementation intervention (4 × 5 g/day in the first week followed 5 g/day in the next five weeks) on maximal-intensity and endurance performance in female soccer players during in-season training and found that creatine produced improved results in the jumps and repeated sprinting performance tests [270].

In events where a supercompensation protocol is possible or desirable (i.e., competitions that take place in just one or a few days), creatine ingestion (20 g/day for six days) can augment dietary carbohydrate mediated muscle glycogen supercompensation during the initial 24 h of recovery (sustaining it on the next five days) following prolonged exhaustive exercise [274].

Nevertheless, creatine does not always produce improved performance results, whether using an acute [275] or a chronic supplementation protocol [276]. Non-responders typically have higher preload levels of creatine and phosphocreatine, less type II muscle fibers, small preload muscle cross-sectional area, and lower fat-free mass [277].

On the methodology of creatine supplementation there are various issues that should be addressed. For example, creatine supplementation seems to be most effective when taken after exercise, both on healthy older adults or healthy young adults, at a dose of 0.1 g·kg^−1^ or around 5 g [278,279]. The ideal frequency of weekly ingestion is not known but on physically active university students there was no difference between a two or a three day/week intake schedule [280]. Nevertheless, professional soccer players often train/play 5–6 days per week and should supplement accordingly, or at least after high intensity training or every match. Creatine ingested in combination with CHO substantially increased muscle creatine accumulation compared with the ingestion of creatine alone [281] but a combination of 50 g of CHO and 50 g of protein seem to be as effective as an isolated dose of 100 g of CHO [282]. Otherwise, a recent review on creatine supplementation and lower limb strength performance found that this supplement is effective for exercise with a duration of less than 3 min, independent of population characteristic, training protocols (including the non-necessity of a loading phase) and supplementary doses and duration [283]. The same study states that creatine monohydrate is still the most used form of creatine supplementation. Finally, long-term creatine use does not appear to result in adverse health effects, at least while consuming the most popular creatine forms [246,284].

#### 6.6.4. Nitrate

Dietary nitrate supplementation is known for its capacity for reducing the oxygen cost of submaximal exercise [285]. The ingestion of nitrate leads to an increment of plasma nitrite concentrations and, consequently, to an increased production of nitric oxide [286]. Nitric oxide has several metabolic and vascular effects that seem to contribute to a better exercise efficiency [286]. Dietary nitrate is available in the form of beetroot shots, nitrate-containing gels and bars [15]. The ingestion of nitrate-rich foods such as spinach, lettuce and arugula might be an alternative to supplementation [287]. 

Although the use of nitrate became popular on soccer, few studies have investigated the effectiveness of nitrates specifically on soccer performance. Wylie and collaborators [247] investigated the intake of 490 mL of nitrate-rich beetroot juice concentrated or placebo over ≈30 h preceding the completion of a Yo-Yo intermittent recovery level 1 test (IR1) by male recreational team-sport players. The supplementation protocol consisted of the ingestion of 2 × 70 mL (2 × ≈4.1 mmol of nitrate) in the morning and 2 × 70 mL in the evening. On each experimental day, subjects consumed a further 2 × 70 mL 2.5 h prior to and 1 × 70 mL 1.5 h prior to the start of the exercise protocol. Performance in the Yo-Yo IR1 test was 4.2% greater with the beetroot juice compared to placebo. More recently, Thompson and collaborators [248] studied the intake of 70 mL beetroot juice/day (≈6.4 mmol of nitrate) or placebo for five days by male team-sport players. On day 5, subjects completed a series of maximal 20-m sprints followed by the Yo-To IR1; cognitive tasks were completed prior to, during and immediately following the Yo-Yo IR1. The distance covered in the Yo-Yo IR1 test improved by 3.9% compared to placebo. The reaction time to the cognitive tasks was 4.7% shorter in the beetroot juice group than in the placebo group at rest but not during the Yo-Yo IR1. In another study, [288], improvements in total work done during prolonged intermittent exercise and in reaction time of response to cognitive tasks in the second half of the intermittent exercise protocol were also achieved with the ingestion of a larger daily dosage (140 mL beetroot juice; 12.8 mmol of nitrate) during a larger period of time (7 days). 

Nitrate seems to be a promising dietary supplement in the context of sport. Nevertheless, further research on high-intensity intermittent team sports such as soccer with elite athletes is necessary. 

#### 6.6.5. Sodium Bicarbonate

Although there is a rationale to use alkalizing agents such as sodium bicarbonate on soccer, to our knowledge, few studies have been performed regarding this issue. Saunders and collaborators [289] investigated the acute supplementation of sodium bicarbonate (0.3 g/kg–0.2 g/kg 4 h before exercise and 0.1 g/kg 2 h before exercise) or placebo on three sets of 5 × 6 s repeated sprints performed during a football specific intermittent treadmill protocol performed in hypoxia (15.5% O_2_). In this study, the supplementation with sodium bicarbonate did not improve repeated sprints performance. In another study, [290] with male rugby players, the ingestion of 0.3 g/kg of sodium bicarbonate 65 min before a 25-min warm-up followed by 9 min of high-intensity rugby-specific training followed by a rugby-specific repeated-sprint test, increased blood HCO_3^−^_ concentration and attenuated the decline in blood pH compared with placebo but did not significantly improve exercise performance. Contrarily, in a recent study [291], high-intensity intermittent exercise performance was improved by the intake of 0.4 g/kg of sodium bicarbonate before the Yo-Yo intermittent recovery test level 2. The sodium bicarbonate was evenly distributed in ~25 gelatin capsules, with one fifth taken at 90, 80, 70, 60 and 50 min prior to exercise in order to avoid stomach discomfort. 

Nevertheless, a review from Bishop on the dietary supplements for team-sport performance [249] concluded that based on several studies on running and cycling sprint performances, the ingestion of sodium bicarbonate is likely to improve both repeated- and intermittent-sprint performance. More research is needed to confirm the possible ergogenic effect of sodium bicarbonate on soccer.

## 7. Conclusions

Finding the “sweet spot” between training adaptations, performance and sound recovery is challenging. Nutrition plays an important role in this process, along with the management of training and match loads as well as other performance and recovery strategies (e.g., ice baths, massage, cryotherapy, software for soccer analysis and the development of better sports equipment, among others). In fact, research shows us that the potential benefits of a sound nutritional support seem unequivocal for soccer performance and recovery. Today, most soccer teams are concerned with their players’ nutritional habits, and generally provide them with detailed nutritional individual plans. Here, a food first approach is of paramount importance but when an adequate nutrition is already in practice, the sensible use of evidence-based ergogenic supplements may further optimize a player’s performance.

In this review, we have provided detailed information on the most recent recommendations for macro and micronutrient intakes, as well as hydration and selected performance-enhancing supplements. Whenever possible, we also provided relevant information on the timing of ingestion, according to a soccer player’s condition. In particular, readers can find nutritional guidelines for daily nutritional intakes, pre-, peri- and post-match/exercise and competition. We also cover the more recent issue of periodized nutrition, a newer strategy to improve soccer performance through manipulation of macronutrient intake in relation to muscle nutrient needs and metabolic adaptations.

Future research should investigate if these recommendations apply for women and younger soccer players, since they may display different nutritional intake needs. Nevertheless, and while these recommendations seem to be the most relevant and up to date, we should recall that nutrition is an evolving science and that future research will soon strengthen some of the prevailing practices, possibly discard others and likely introduce innovative and improved nutritional strategies. All of those will most certainly have a desired and decisive impact on the optimization of soccer performance.

## Figures and Tables

**Table 1:** Recommended intakes for selected macronutrients in different situations (includes hydration).

Situation	Recommendations		Practical Considerations	Reference
Daily requirements	CHO: 5–10 g/kg/day		Adjust to the individual nutritional goals and periodize according to the needs of daily training sessions; consider low CHO availability in lower intensity training sessions to improve the metabolic effects of exercise.	[67]
	Protein: 1.2–2.0 g/kg/day		Choose higher range in pre-season, after injury, after high intensity training and/or when in a low energy budget.	[12]
	Hydration: consume sufficient fluids before, during, and after exercise to sustain health and performance; daily monitoring of first-voiding urine color is a practical hydration status assessment tool.			[68]
	Fat: an intake of at least 20% of total energy intake from fat is advised.			[12]
Pre-training and matches	CHO: 1–4 g/kg		Adjust according to the session needs and individual tolerance; Choose lower range if restricting calories.	[69,70]
	Protein: 0.25–0.4 g/kg		Choose an amount near the higher range when in a low energy budget and/or before resistance training.	[71]
	Hydration: ~5–7 mL/kg—at least 4 h before the exercise task. If urine is not produced, or urine is dark or highly concentrated: ~3–5 mL/kg—about 2 h before the event.		Enhancing palatability of the ingested fluid will help to promote fluid consumption. The preferred water temperature is often between 15 and 21 °C.	[72]
During training	CHO	Light training: no need, provided sufficient pre-training HCO was consumed.		[70,73]
		Hard training/Two sessions a day: 30–60 g/kg	Provide the highest amount when performing an afternoon session <8 h after hard morning session; consider the addition of a small amount of protein to the CHO solution.	
	Hydration: sufficient fluids must be consumed to avoid (a) losing more than 2% of initial BW and (b) weight gain.		Athletes must be aware their sweat rates. The addition of small amounts of salt must be considered during prolonged training sessions in the heat.	[72]
After training	CHO	Light training: follow food plan to ensure daily needs are met		[16,73,74]
		Hard training/Two sessions a day: 1.0–1.2 g CHO/kg/h	Start refueling immediately after training; check for individual glycemic response to ensure high CHO availability.	
		Protein: 0.25–0.4 g/kg	Choose an amount near the higher range after high intensity and/or resistance training.	[71]
		Hydration: ingest 125–150% of fluids lost.	Salty foods and drinks may help retaining ingested water. Drink regularly rather than one large bolus.	[12]
During competition	CHO: 30–60 g/h or small amounts or mouth rinsing if the athlete is going to compete for a short amount of time (30 min–1 h).		Small sips or rinsing of sports drinks. Test in training before practicing in matches.	[16,73]
	Hydration: ad libitum		Especially relevant when pre-match hydration status is inadequate.	[72]
After competition	CHO	72 h or less until next match: 1–1.2 g CHO/kg/h OR 0.8 g CHO/kg plus 0.4 g protein/kg/h		[16,70]
		More than 72 h until next match: ad libitum, provided daily needs are met		
		Private events, single matches: ad libitum	Eat and drink taking into account individual nutrition and body composition goals and the next competitive commitments.	
	Protein: similar to post-training			[71]
	Hydration: ad libitum			[12]

Abbreviations: CHO, carbohydrates.

**Table 2:** Sweat losses, sweat rates and intake recommendations for an adequate hydration in soccer.

N	Environmental Conditions	Duration	Mean Sweat Losses (mL)	Sweat Rates (mL/h)	Fluid Intake (mL)	Dehydration (% BML)	Reference
**Training sessions**							
**24 PP**	T: 24–49 °C RL: 46–64%	90 min	2033 ± 413	1355 ± 275	971 ± 303	1.37 ± 0,54	[136]
**26 PP**	T: 32 ± 3 °C RL: 20 ± 5%	90 min	2193 ± 365	Not reported	972 ± 335	1.59 ± 0.61	[135]
**17 PP**	T: 5 ± 0.7 °C RL: 81 ± 6%	100 min	1690 ± 450	1130 + 300	423 ± 215	1.62 ± 0.55	[137]
**Match play (including simulation)**							
**17 PP**	T: 35 ± 1 °C RH: 35 ± 4	90 min	4448 ± 1216	1483 + 362	1948 ± 954	3.4 ± 1.1	[132]
**13 PP**	T: 27 ± 0.1 °C R: 65 ± 7%	100 min	2600 ± 600	Not reported	1666 ± 333	3.4 ± 0.7	[138]
**20 PP**	T: 6–8 °C RL: 50–60%	90 min	1680 ± 400	Not reported	840 ± 470	1.1 ± 0.6	[129]
**20 PP**	T: 29 ± 1.1 °C RH: 64 ± 4.2%.	90 min	2360 ± 530	866 ± 319	1265.00 ± 505.45	1.35 ± 0.87	[139]

PP: professional players; T: environmental temperature; RL: Relative humidity.

**Table 3:** Practical recommendations in selected performance dietary supplements.

Supplement	Rational for Intake	Protocol and Practical Recommendations	References
Beta-alanine	Increase in muscle buffer capacity. Delay in the onset of muscular fatigue. Facilitated recovery during repeated bouts of high-intensity exercise.	4–6 g/day, for at least 2–4 weeks. Attenuate paresthesia by using divided lower doses (1.6 g) or using a sustained-release formula and avoid intake before a match.	[242,243]
Caffeine	Improve cognitive and skill performance. Decrease perceived exertion.	3–6 mg/kg 60 min before kick-off.	[15,70,244,245],
Creatine	Maintenance of intracellular levels of adenosine triphosphate. Improvement of power, strength and muscle mass.	0.1 g/kg or 5 g/day after training/match. Add 100 g CHO or 50 g CHO + 50 g protein for optimal absorption.	[245,246]
Nitrate	Decrease oxygen cost of submaximal exercise.	6–8 mmol/day for 2–5 days before a match and 90 min before kick-off.	[15,247,248]
Sodium bicarbonate	Greater extracellular buffer concentration increasing H^+^ efflux from the muscles into the blood.	0.2–0.3 g/kg ingested 60–120 min before exercise. May cause gastrointestinal side-effects.	[249] Note: recommendations based on limited evidence

## References

[1] Giulianotti R., Robertson R. (2004). The globalization of football: A study in the glocalization of the ′serious life’. Br. J. Soc..

[2] Di Salvo V., Gregson W., Atkinson G., Tordoff P., Drust B. (2009). Analysis of high intensity activity in Premier League soccer. Int. J. Sports Med..

[3] Bush M., Barnes C., Archer D.T., Hogg B., Bradley P.S. (2015). Evolution of match performance parameters for various playing positions in the English Premier League. Hum. Mov. Sci..

[4] Strudwick A.J., Williams M.A. (2013). Contemporary Issues in the Physical Preparation of Elite Players. Science and Soccer: Developing Elite Performers.

[5] Williams A.M., Lee D., Reilly T. (1999). A Quantitative Analysis of Matches Played in the 1991–1992 and 1997–1998 Seasons.

[6] Anderson L., Orme P., Di Michele R., Close G.L., Morgans R., Drust B., Morton J.P. (2016). Quantification of training load during one-, two- and three-game week schedules in professional soccer players from the English Premier League: Implications for carbohydrate periodisation. J. Sports Sci..

[7] Dupont G., Nedelec M., McCall A., McCormack D., Berthoin S., Wisløff U. (2010). Effect of 2 soccer matches in a week on physical performance and injury rate. Am. J. Sports Med..

[8] Nedelec M., Mc Call A., Carling C., Legall F., Berthoin S., Dupont G. (2012). Recovery in Soccer Part I—Post-Match Fatigue and Time Course of Recovery. Sports Med..

[9] Silva J.R., Nassis G.P., Rebelo A. (2015). Strength training in soccer with a specific focus on highly trained players. Sports Med. Open.

[10] Clarke J.D., Carré M.J. (2010). Improving the performance of soccer boots on artificial and natural soccer surfaces. Procedia Eng..

[11] Carling C., Williams A.M., Reilly T. (2006). Handbook of Soccer Match Analysis: A Systematic Approach to Improving Performance. J. Sports Sci. Med..

[12] Thomas D.T., Erdman K.A., Burke L.M. (2016). American College of Sports Medicine Joint Position Statement. Nutrition and Athletic Performance. Med. Sci. Sports Exerc..

[13] Ranchordas M., Strudwick A. (2016). Nutritional Needs. Soccer Science.

[14] Maughan R. (2006). Nutrition and Football: The FIFA/FMARC Consensus on Sports Nutrition.

[15] Strudwick A. (2016). Soccer Science.

[16] Burke L., Deakin V. (2015). Clinical Sports Nutrition.

[17] Mark Williams A. (2013). Science and Soccer: Developing Elite Performers.

[18] Bangsbo J. (1994). Energy demands in competitive soccer. J. Sports Sci..

[19] Bloomfield J., Polman R., O’Donoghue P. (2007). Physical Demands of Different Positions in FA Premier League Soccer. J. Sports Sci. Med..

[20] Mohr M., Krustrup P., Bangsbo J. (2003). Match performance of high-standard soccer players with special reference to development of fatigue. J. Sports Sci..

[21] Bangsbo J. (2014). Physiological demands of Football. Sports Sci. Exch..

[22] Carling C., Bloomfield J. (2010). The effect of an early dismissal on player work-rate in a professional soccer match. J. Sci. Med. Sport.

[23] Carling C., Court M., Williams M.A. (2013). Match and motion analysis. Science and Soccer: Developing Elite Performers.

[24] Mohr M., Krustrup P., Bangsbo J. (2005). Fatigue in soccer: A brief review. J. Sports Sci..

[25] Mohr M., Krustrup P., Nybo L., Nielsen J.J., Bangsbo J. (2004). Muscle temperature and sprint performance during soccer matches—Beneficial effect of re-warm-up at half-time. Scand. J. Med. Sci. Sports.

[26] Krustrup P., Mohr M., Steensberg A., Bencke J., Kjaer M., Bangsbo J. (2006). Muscle and blood metabolites during a soccer game: Implications for sprint performance. Med. Sci. Sports Exerc..

[27] Di Salvo V., Baron R., Tschan H., Calderon Montero F.J., Bachl N., Pigozzi F. (2007). Performance characteristics according to playing position in elite soccer. Int. J. Sports Med..

[28] Di Salvo V., Pigozzi F., González-Haro C., Laughlin M.S., De Witt J.K. (2013). Match performance comparison in top English soccer leagues. Int. J. Sports Med..

[29] Carling C., Le Gall F., Dupont G. (2012). Analysis of repeated high-intensity running performance in professional soccer. J. Sports Sci..

[30] Dupont G., McCall A., Strudwick T. (2016). Targeted systems of the body for training. Soccer Science.

[31] Duthie G., Pyne D., Hooper S. (2003). Applied physiology and game analysis of rugby union. Sports Med..

[32] Reilly T.S.N., Snell P., Williams C. (1990). Physiology of Sports.

[33] Rodriguez N.R., Di Marco N.M., Langley S. (2009). Nutrition and athletic performance. Med. Sci. Sports Exerc..

[34] Reilly T., Reilly T. (1996). Fitness assessment. Science and Soccer.

[35] Gabbett T.J. (2005). Science of rugby league football: A review. J. Sports Sci..

[36] Silvestre R., Kraemer W.J., West C., Judelson D.A., Spiering B.A., Vingren J.L., Hatfield D.L., Anderson J.M., Maresh C.M. (2006). Body composition and physical performance during a National Collegiate Athletic Association Division I men’s soccer season. J. Strength Cond. Res..

[37] Lago-Penas C., Casais L., Dellal A., Rey E., Dominguez E. (2011). Anthropometric and physiological characteristics of young soccer players according to their playing positions: Relevance for competition success. J. Strength Cond. Res..

[38] Nikolaidis P.T., Vassilios Karydis N. (2011). Physique and Body Composition in Soccer Players across Adolescence. Asian J. Sports Med..

[39] Nikolaidis P.T. (2012). Elevated body mass index and body fat percentage are associated with decreased physical fitness in soccer players aged 12–14 years. Asian J. Sports Med..

[40] Nikolaidis P.T. (2012). Physical fitness is inversely related with body mass index and body fat percentage in soccer players aged 16–18 years. Med. Pregl..

[41] Brocherie F., Girard O., Forchino F., Al Haddad H., Dos Santos G.A., Millet G.P. (2014). Relationships between anthropometric measures and athletic performance, with special reference to repeated-sprint ability, in the Qatar national soccer team. J. Sports Sci..

[42] Nikolaidis P., Dellal A., Torres-Luque G., Ingebrigtsen J. (2015). Determinants of acceleration and maximum speed phase of repeated sprint ability in soccer players: A cross-sectional study. Sci. Sports.

[43] Nikolaidis P.T., Ruano M.A., de Oliveira N.C., Portes L.A., Freiwald J., Lepretre P.M., Knechtle B. (2016). Who runs the fastest? Anthropometric and physiological correlates of 20 m sprint performance in male soccer players. Res. Sports Med..

[44] Silvestre R., West C., Maresh C.M., Kraemer W.J. (2006). Body composition and physical performance in men’s soccer: A study of a National Collegiate Athletic Association Division I team. J. Strength Cond. Res..

[45] Reilly T., George K., Marfell-Jones M., Scott M., Sutton L., Wallace J.A. (2009). How well do skinfold equations predict percent body fat in elite soccer players?. Int. J. Sports Med..

[46] Wittich A., Oliveri M.B., Rotemberg E., Mautalen C. (2001). Body composition of professional football (soccer) players determined by dual X-ray absorptiometry. J. Clin. Densitom..

[47] Matkovic B.R., Misigoj-Durakovic M., Matkovic B., Jankovic S., Ruzic L., Leko G., Kondric M. (2003). Morphological differences of elite Croatian soccer players according to the team position. Coll. Antropol..

[48] Ozcakar L., Cetin A., Kunduracyolu B., Ulkar B. (2003). Comparative body fat assessment in elite footballers. Br. J. Sports Med..

[49] Hencken C., White C. (2006). Anthropometric assessment of Premiership soccer players in relation to playing position. Eur. J. Sport Sci..

[50] Fredericson M., Chew K., Ngo J., Cleek T., Kiratli J., Cobb K. (2007). Regional bone mineral density in male athletes: A comparison of soccer players, runners and controls. Br. J. Sports Med..

[51] Melchiorri G., Monteleone G., Andreoli A., Calla C., Sgroi M., De Lorenzo A. (2007). Body cell mass measured by bioelectrical impedance spectroscopy in professional football (soccer) players. J. Sports Med. Phys. Fit..

[52] Sutton L., Scott M., Wallace J., Reilly T. (2009). Body composition of English Premier League soccer players: Influence of playing position, international status, and ethnicity. J. Sports Sci..

[53] Carling C., Orhant E. (2010). Variation in body composition in professional soccer players: Interseasonal and intraseasonal changes and the effects of exposure time and player position. J. Strength Cond. Res..

[54] Milsom J., Naughton R., O’Boyle A., Iqbal Z., Morgans R., Drust B., Morton J.P. (2015). Body composition assessment of English Premier League soccer players: A comparative DXA analysis of first team, U21 and U18 squads. J. Sports Sci..

[55] Reilly T., Bangsbo J., Franks A. (2000). Anthropometric and physiological predispositions for elite soccer. J. Sports Sci..

[56] Arnason A., Sigurdsson S.B., Gudmundsson A., Holme I., Engebretsen L., Bahr R. (2004). Physical fitness, injuries, and team performance in soccer. Med. Sci. Sports Exerc..

[57] Milanese C., Cavedon V., Corradini G., De Vita F., Zancanaro C. (2015). Seasonal DXA-measured body composition changes in professional male soccer players. J. Sports Sci..

[58] Devlin B.L., Kingsley M., Leveritt M.D., Belski R. (2016). Seasonal changes in soccer players’ body composition and dietary intake practices. J. Strength Cond. Res..

[59] Ackland T.R., Lohman T.G., Sundgot-Borgen J., Maughan R.J., Meyer N.L., Stewart A.D., Muller W. (2012). Current status of body composition assessment in sport: Review and position statement on behalf of the ad hoc research working group on body composition health and performance, under the auspices of the I.O.C. Medical Commission. Sports Med..

[60] Reilly T., Thomas V. (1979). Estimated daily energy expenditures of professional association footballers. Ergonomics.

[61] Rico-Sanz J. (1998). Body composition and nutritional assessments in soccer. Int. J. Sport Nutr..

[62] Osgnach C., Poser S., Bernardini R., Rinaldo R., di Prampero P.E. (2010). Energy cost and metabolic power in elite soccer: A new match analysis approach. Med. Sci. Sports Exerc..

[63] Anderson L., Orme P., Naughton R.J., Close G.L., Milsom J., Rydings D., O’Boyle A., Di Michele R., Louis J., Hambley C. (2017). Energy Intake and Expenditure of Professional Soccer Players of the English Premier League: Evidence of Carbohydrate Periodization. Int. J. Sport Nutr. Exerc. Metab..

[64] Bangsbo J., Mohr M., Krustrup P. (2006). Physical and metabolic demands of training and match-play in the elite football player. J. Sports Sci..

[65] Bettonviel A.E., Brinkmans N.Y., Russcher K., Wardenaar F.C., Witard O.C. (2016). Nutritional Status and Daytime Pattern of Protein Intake on Match, Post-Match, Rest and Training Days in Senior Professional and Youth Elite Soccer Players. Int. J. Sport Nutr. Exerc. Metab..

[66] Rampinini E., Impellizzeri F.M., Castagna C., Coutts A.J., Wisloff U. (2009). Technical performance during soccer matches of the Italian Serie A league: effect of fatigue and competitive level. J. Sci. Med. Sport.

[67] Burke L.M., Hawley J.A., Wong S.H., Jeukendrup A.E. (2011). Carbohydrates for training and competition. J. Sports Sci..

[68] Maughan R.J., Shirreffs S.M. (2008). Development of individual hydration strategies for athletes. Int. J. Sport Nutr. Exerc. Metab..

[69] Balsom P.D., Wood K., Olsson P., Ekblom B. (1999). Carbohydrate intake and multiple sprint sports: With special reference to football (soccer). Int. J. Sports Med..

[70] Russell M., Kingsley M. (2014). The efficacy of acute nutritional interventions on soccer skill performance. Sports Med..

[71] Morton R.W., McGlory C., Phillips S.M. (2015). Nutritional interventions to augment resistance training-induced skeletal muscle hypertrophy. Front. Physiol..

[72] Sawka M.N., Burke L.M., Eichner E.R., Maughan R.J., Montain S.J., Stachenfeld N.S. (2007). American College of Sports Medicine position stand. Exercise and fluid replacement. Med. Sci. Sports Exerc..

[73] Baker L.B., Rollo I., Stein K.W., Jeukendrup A.E. (2015). Acute Effects of Carbohydrate Supplementation on Intermittent Sports Performance. Nutrients.

[74] Burke L.M., van Loon L.J.C., Hawley J.A. (2016). Post-exercise muscle glycogen resynthesis in humans. J. Appl. Physiol..

[75] Hawley J.A., Morton J.P. (2014). Ramping up the signal: Promoting endurance training adaptation in skeletal muscle by nutritional manipulation. Clin. Exp. Pharmacol. Physiol..

[76] Marquet L.-A., Hausswirth C., Molle O., Hawley J., Burke L., Tiollier E., Brisswalter J. (2016). Periodization of Carbohydrate Intake: Short-Term Effect on Performance. Nutrients.

[77] Marquet L.A., Brisswalter J., Louis J., Tiollier E., Burke L.M., Hawley J.A., Hausswirth C. (2016). Enhanced Endurance Performance by Periodization of Carbohydrate Intake: “Sleep Low” Strategy. Med. Sci. Sports Exerc..

[78] Chryssanthopoulos C., Williams C., Nowitz A., Bogdanis G. (2004). Skeletal muscle glycogen concentration and metabolic responses following a high glycaemic carbohydrate breakfast. J. Sports Sci..

[79] Wright D.A., Sherman W.M., Dernbach A.R. (1991). Carbohydrate feedings before, during, or in combination improve cycling endurance performance. J. Appl. Physiol..

[80] Neufer P.D., Costill D.L., Flynn M.G., Kirwan J.P., Mitchell J.B., Houmard J. (1987). Improvements in exercise performance: Effects of carbohydrate feedings and diet. J. Appl. Physiol..

[81] Sherman W.M., Brodowicz G., Wright D.A., Allen W.K., Simonsen J., Dernbach A. (1989). Effects of 4 h preexercise carbohydrate feedings on cycling performance. Med. Sci. Sports Exerc..

[82] Jeukendrup A.E., Killer S.C. (2010). The myths surrounding pre-exercise carbohydrate feeding. Ann. Nutr. Metab..

[83] Sherman W.M., Peden M.C., Wright D.A. (1991). Carbohydrate feedings 1 h before exercise improves cycling performance. Am. J. Clin. Nutr..

[84] McInerney P., Lessard S.J., Burke L.M., Coffey V.G., Lo Giudice S.L., Southgate R.J., Hawley J.A. (2005). Failure to repeatedly supercompensate muscle glycogen stores in highly trained men. Med. Sci. Sports Exerc..

[85] Harper L.D., Hunter R., Parker P., Goodall S., Thomas K., Howatson G., West D.J., Stevenson E., Russell M. (2016). Test-Retest Reliability of Physiological and Performance Responses to 120 Minutes of Simulated Soccer Match Play. J. Strength Cond. Res..

[86] Goedecke J.H., White N.J., Chicktay W., Mahomed H., Durandt J., Lambert M.I. (2013). The effect of carbohydrate ingestion on performance during a simulated soccer match. Nutrients.

[87] Nicholas C.W., Williams C., Lakomy H.K., Phillips G., Nowitz A. (1995). Influence of ingesting a carbohydrate-electrolyte solution on endurance capacity during intermittent, high-intensity shuttle running. J. Sports Sci..

[88] Baker L.B., Nuccio R.P., Jeukendrup A.E. (2014). Acute effects of dietary constituents on motor skill and cognitive performance in athletes. Nutr. Rev..

[89] Phillips S.M., Sproule J., Turner A.P. (2011). Carbohydrate Ingestion during Team Games Exercise. Sports Med..

[90] Bandelow S., Maughan R., Shirreffs S., Ozgunen K., Kurdak S., Ersoz G., Binnet M., Dvorak J. (2010). The effects of exercise, heat, cooling and rehydration strategies on cognitive function in football players. Scand. J. Med. Sci. Sports.

[91] Alghannam A.F. (2011). Carbohydrate-protein ingestion improves subsequent running capacity towards the end of a football-specific intermittent exercise. Appl. Physiol. Nutr. Metab..

[92] Jeukendrup A.E., Chambers E.S. (2010). Oral carbohydrate sensing and exercise performance. Curr. Opin. Clin. Nutr. Metab. Care.

[93] Jeukendrup A.E. (2013). Oral carbohydrate rinse: Placebo or beneficial?. Curr. Sports Med. Rep..

[94] Chambers E.S., Bridge M.W., Jones D.A. (2009). Carbohydrate sensing in the human mouth: Effects on exercise performance and brain activity. J. Physiol..

[95] de Ataide e Silva T., Di Cavalcanti Alves de Souza M.E., de Amorim J.F., Stathis C.G., Leandro C.G., Lima-Silva A.E. (2013). Can carbohydrate mouth rinse improve performance during exercise? A systematic review. Nutrients.

[96] Bangsbo J., Gollnick P.D., Graham T.E., Saltin B. (1991). Substrates for muscle glycogen synthesis in recovery from intense exercise in man. J. Physiol..

[97] Ivy J.L., Lee M.C., Brozinick J.T., Reed M.J. (1988). Muscle glycogen storage after different amounts of carbohydrate ingestion. J Appl. Physiol..

[98] Zeevi D., Korem T., Zmora N., Israeli D., Rothschild D., Weinberger A., Ben-Yacov O., Lador D., Avnit-Sagi T., Lotan-Pompan M. (2015). Personalized Nutrition by Prediction of Glycemic Responses. Cell.

[99] Betts J.A., Williams C. (2010). Short-term recovery from prolonged exercise: Exploring the potential for protein ingestion to accentuate the benefits of carbohydrate supplements. Sports Med..

[100] Tarnopolsky M.A., Zawada C., Richmond L.B., Carter S., Shearer J., Graham T., Phillips S.M. (2001). Gender differences in carbohydrate loading are related to energy intake. J. Appl. Physiol..

[101] Devlin B.L., Leveritt M.D., Kingsley M., Belski R. (2016). Dietary Intake, Body Composition and Nutrition Knowledge of Australian Football and Soccer Players: Implications for Sports Nutrition Professionals in Practice. Int. J. Sport Nutr. Exerc. Metab..

[102] Naughton R.J., Drust B., O’Boyle A., Morgans R., Abayomi J., Davies I.G., Morton J.P., Mahon E. (2016). Daily Distribution of Carbohydrate, Protein and Fat Intake in Elite Youth Academy Soccer Players Over a 7-Day Training Period. Int. J. Sport Nutr. Exerc. Metab..

[103] Briggs M.A., Cockburn E., Rumbold P.L., Rae G., Stevenson E.J., Russell M. (2015). Assessment of Energy Intake and Energy Expenditure of Male Adolescent Academy-Level Soccer Players during a Competitive Week. Nutrients.

[104] Moore D.R., Robinson M.J., Fry J.L., Tang J.E., Glover E.I., Wilkinson S.B., Prior T., Tarnopolsky M.A., Phillips S.M. (2009). Ingested protein dose response of muscle and albumin protein synthesis after resistance exercise in young men. Am. J. Clin. Nutr..

[105] Moore D.R., Churchward-Venne T.A., Witard O., Breen L., Burd N.A., Tipton K.D., Phillips S.M. (2015). Protein ingestion to stimulate myofibrillar protein synthesis requires greater relative protein intakes in healthy older versus younger men. J. Gerontol. Ser. A Biol. Sci. Med. Sci..

[106] Macnaughton L.S., Wardle S.L., Witard O.C., McGlory C., Hamilton D.L., Jeromson S., Lawrence C.E., Wallis G.A., Tipton K.D. (2016). The response of muscle protein synthesis following whole-body resistance exercise is greater following 40 g than 20 g of ingested whey protein. Physiol. Rep..

[107] Esmarck B., Andersen J., Olsen S., Richter E., Mizuno M., Kjaer M. (2001). Timing of postexercise protein intake is important for muscle hypertrophy with resistance training in elderly humans. J. Physiol..

[108] Areta J.L., Burke L.M., Ross M.L., Camera D.M., West D.W.D., Broad E.M., Jeacocke N.A., Moore D.R., Stellingwerff T., Phillips S.M. (2013). Timing and distribution of protein ingestion during prolonged recovery from resistance exercise alters myofibrillar protein synthesis. J. Physiol..

[109] Mamerow M.M., Mettler J.A., English K.L., Casperson S.L., Arentson-Lantz E., Sheffield-Moore M., Layman D.K., Paddon-Jones D. (2014). Dietary protein distribution positively influences 24-h muscle protein synthesis in healthy adults. J. Nutr..

[110] Tipton K., Rasmussen B., Miller S., Wolf S., Owens-Stovall S., Petrini B., Wolfe R. (2001). Timing of amino acid-carbohydrate ingestion alters anabolic response of muscle to resistance exercise. Am. J. Physiol. Endocrinol. Metab..

[111] Res P.T., Groen B., Pennings B., Beelen M., Wallis G.A., Gijsen A.P., Senden J.M.G., Van Loon L.J.C. (2012). Protein ingestion before sleep improves postexercise overnight recovery. Med. Sci. Sports Exerc..

[112] Snijders T., Res P.T., Smeets J.S.J., Van Vliet S., Van Kranenburg J., Maase K., Kies A.K., Verdijk L.B., Van Loon L.J.C. (2015). Protein ingestion before sleep increases muscle mass and strength gains during prolonged resistance-type exercise training in healthy young men. J. Nutr..

[113] Van Loon L.J. (2014). Is there a need for protein ingestion during exercise?. Sports Med..

[114] Romagnoli M., Sanchis-Gomar F., Alis R., Risso-Ballester J., Bosio A., Graziani R.L., Rampinini E. (2016). Changes in muscle damage, inflammation, and fatigue-related parameters in young elite soccer players after a match. J. Sports Med. Phys. Fit..

[115] Beelen M., Koopman R., Gijsen A.P., Vandereyt H., Kies A.K., Kuipers H., Saris W.H., van Loon L.J. (2008). Protein coingestion stimulates muscle protein synthesis during resistance-type exercise. Am. J. Physiol. Endocrinol. Metab..

[116] Beelen M., Tieland M., Gijsen A.P., Vandereyt H., Kies A.K., Kuipers H., Saris W.H., Koopman R., van Loon L.J. (2008). Coingestion of carbohydrate and protein hydrolysate stimulates muscle protein synthesis during exercise in young men, with no further increase during subsequent overnight recovery. J. Nutr..

[117] Phillips S.M., Van Loon L.J. (2011). Dietary protein for athletes: From requirements to optimum adaptation. J. Sports Sci..

[118] Sousa M., Teixeira V.H., Soares J. (2014). Dietary strategies to recover from exercise-induced muscle damage. Int. J. Food Sci. Nutr..

[119] Burke L.M. (2015). Re-examining high-fat diets for sports performance: Did we call the ‘nail in the coffin’ too soon?. Sports Med..

[120] Potgieter S. (2013). Sport nutrition: A review of the latest guidelines for exercise and sport nutrition from the American College of Sport Nutrition, the International Olympic Committee and the International Society for Sports Nutrition. S. Afr. J. Clin. Nutr..

[121] Kreider R.B., Wilborn C.D., Taylor L., Campbell B., Almada A.L., Collins R., Cooke M., Earnest C.P., Greenwood M., Kalman D.S. (2010). ISSN exercise & sport nutrition review: Research & recommendations. J. Int. Soc. Sports Nutr..

[122] Simopoulos A.P. (2007). Omega-3 fatty acids and athletics. Curr. Sports Med. Rep..

[123] Peoples G.E., McLennan P.L., Howe P., Groeller H. Fish oil reduces apparent myocardial oxygen consumption in trained cyclists but does not change time to fatigue. Presented at the Fourth International Conference on Nutrition and Fitness.

[124] Göransson U., Karlsson J., Rønneberg R., Rasmusson M., Toomey W.A. (1997). The ‘Are’ Sport Nutratherapy Program: The rationale for food supplements in sports medicine. World Rev. Nutr. Diet..

[125] Jeukendrup A.E., Saris W.H., Schrauwen P., Brouns F., Wagenmakers A.J. (1995). Metabolic availability of medium-chain triglycerides coingested with carbohydrates during prolonged exercise. J. Appl. Physiol..

[126] Burke L.M., Collier G.R., Beasley S.K., Davis P.G., Fricker P.A., Heeley P., Walder K., Hargreaves M. (1995). Effect of coingestion of fat and protein with carbohydrate feedings on muscle glycogen storage. J. Appl. Physiol..

[127] Roy B.D., Tarnopolsky M.A. (1998). Influence of differing macronutrient intakes on muscle glycogen resynthesis after resistance exercise. J. Appl. Physiol..

[128] Elliot T., Cree M., Sanford A., Wolfe R., Tipton K. (2006). Milk ingestion stimulates net muscle protein synthesis following resistance exercise. Med. Sci. Sports Exerc..

[129] Maughan R.J., Watson P., Evans G.H., Broad N., Shirreffs S.M. (2007). Water balance and salt losses in competitive football. Int. J. Sport Nutr. Exerc. Metab..

[130] Jequier E., Constant F. (2010). Water as an essential nutrient: The physiological basis of hydration. Eur. J. Clin. Nutr..

[131] Shapiro Y., Pandolf K.B., Goldman R.F. (1982). Predicting sweat loss response to exercise, environment and clothing. Eur. J. Appl. Physiol. Occup. Physiol..

[132] Aragón-Vargas L.F., Moncada-Jiménez J., Hernández-Elizondo J., Barrenechea A., Monge-Alvarado M. (2009). Evaluation of pre-game hydration status, heat stress, and fluid balance during professional soccer competition in the heat. Eur. J. Sport Sci..

[133] Barr S.I., Costill D.L. (1989). Water: Can the endurance athlete get too much of a good thing?. J. Am. Diet. Assoc..

[134] Sawka M.N., Wenger C.B., Pandolf K.B. (2010). Thermoregulatory Responses to Acute Exercise-Heat Stress and Heat Acclimation. Comprehensive Physiology.

[135] Shirreffs S.M., Aragon-Vargas L.F., Chamorro M., Maughan R.J., Serratosa L., Zachwieja J.J. (2005). The sweating response of elite professional soccer players to training in the heat. Int. J. Sports Med..

[136] Maughan R.J., Merson S.J., Broad N.P., Shirreffs S.M. (2004). Fluid and electrolyte intake and loss in elite soccer players during training. Int. J. Sport Nutr. Exerc. Metab..

[137] Maughan R.J., Shirreffs S.M., Merson S.J., Horswill C.A. (2005). Fluid and electrolyte balance in elite male football (soccer) players training in a cool environment. J. Sports Sci..

[138] Duffield R., McCall A., Coutts A.J., Peiffer J.J. (2012). Hydration, sweat and thermoregulatory responses to professional football training in the heat. J. Sports Sci..

[139] Guttierres A.P.M., Natali A.J., Vianna J.M., Reis V.M., Marins J.C.B. (2011). Dehydration in Soccer Players After a Match in the Heat. Biol. Sport.

[140] Coyle E.F., Montain S.J. (1992). Benefits of fluid replacement with carbohydrate during exercise. Med. Sci. Sports Exerc..

[141] Shirreffs S.M., Maughan R.J. (2000). Rehydration and recovery of fluid balance after exercise. Exerc. Sport Sci. Rev..

[142] Burke L.M. (1997). Fluid balance during team sports. J. Sports Sci..

[143] Cheuvront S.N., Haymes E.M. (2001). Thermoregulation and marathon running: Biological and environmental influences. Sports Med..

[144] Coyle E.F. (2004). Fluid and fuel intake during exercise. J. Sports Sci..

[145] Casa D.J., Clarkson P.M., Roberts W.O. (2005). American College of Sports Medicine roundtable on hydration and physical activity: Consensus statements. Curr. Sports Med. Rep..

[146] Edwards A.M., Mann M.E., Marfell-Jones M.J., Rankin D.M., Noakes T.D., Shillington D.P. (2007). Influence of moderate dehydration on soccer performance: Physiological responses to 45 min of outdoor match-play and the immediate subsequent performance of sport-specific and mental concentration tests. Br. J. Sports Med..

[147] McGregor S.J., Nicholas C.W., Lakomy H.K., Williams C. (1999). The influence of intermittent high-intensity shuttle running and fluid ingestion on the performance of a soccer skill. J. Sports Sci..

[148] Ritz P., Berrut G. (2005). The importance of good hydration for day-to-day health. Nutr. Rev..

[149] Sawka M.N. (2005). Dietary Reference Intakes for Water, Potassium, Sodium, Chloride, and Sulfate.

[150] Nybo L., Nielsen B. (2001). Hyperthermia and central fatigue during prolonged exercise in humans. J. Appl. Physiol..

[151] Sawka M.N., Coyle E.F. (1999). Influence of body water and blood volume on thermoregulation and exercise performance in the heat. Exerc. Sport Sci. Rev..

[152] Cheuvront S.N., Carter R., Montain S.J., Sawka M.N. (2004). Daily body mass variability and stability in active men undergoing exercise-heat stress. Int. J. Sport Nutr. Exerc. Metab..

[153] Cheuvront S.N., Ely B.R., Kenefick R.W., Buller M.J., Charkoudian N., Sawka M.N. (2012). Hydration assessment using the cardiovascular response to standing. Eur. J. Appl. Physiol..

[154] Ely B.R., Sollanek K.J., Cheuvront S.N., Lieberman H.R., Kenefick R.W. (2013). Hypohydration and acute thermal stress affect mood state but not cognition or dynamic postural balance. Eur. J. Appl. Physiol..

[155] Judelson D.A., Maresh C.M., Anderson J.M., Armstrong L.E., Casa D.J., Kraemer W.J., Volek J.S. (2007). Hydration and muscular performance: Does fluid balance affect strength, power and high-intensity endurance?. Sports Med..

[156] Goulet E.D. (2011). Effect of exercise-induced dehydration on time-trial exercise performance: A meta-analysis. Br. J. Sports Med..

[157] Cheuvront S.N., Carter R., Haymes E.M., Sawka M.N. (2006). No effect of moderate hypohydration or hyperthermia on anaerobic exercise performance. Med. Sci. Sports Exerc..

[158] Shirreffs S.M., Maughan R.J. (1997). Whole body sweat collection in humans: An improved method with preliminary data on electrolyte content. J. Appl. Physiol..

[159] Shirreffs S.M., Sawka M.N., Stone M. (2006). Water and electrolyte needs for football training and match-play. J. Sports Sci..

[160] Stofan J.N.D., Horswill C.A., Martin R., Bream T., Murray R. (2001). Sweat and sodium losses in cramp-prone professional football players. Med. Sci. Sports Exerc..

[161] Stofan J.R., Zachwieja J.J., Horswill C.A., Murray R., Anderson S.A., Eichner E.R. (2005). Sweat and sodium losses in NCAA football players: A precursor to heat cramps?. Int. J. Sport Nutr. Exerc. Metab..

[162] Bergeron M.F. (2003). Heat cramps: Fluid and electrolyte challenges during tennis in the heat. J. Sci. Med. Sport.

[163] Shirreffs S.M. (2003). Markers of hydration status. Eur. J. Clin. Nutr..

[164] Pialoux V., Mischler I., Mounier R., Gachon P., Ritz P., Coudert J., Fellmann N. (2004). Effect of equilibrated hydration changes on total body water estimates by bioelectrical impedance analysis. Br. J. Nutr..

[165] Ellis K.J., Wong W.W. (1998). Human hydrometry: Comparison of multifrequency bioelectrical impedance with 2H2O and bromine dilution. J. Appl. Physiol..

[166] Gudivaka R., Schoeller D.A., Kushner R.F., Bolt M.J. (1999). Single- and multifrequency models for bioelectrical impedance analysis of body water compartments. J. Appl. Physiol..

[167] Matthie J.R. (2005). Second generation mixture theory equation for estimating intracellular water using bioimpedance spectroscopy. J. Appl. Physiol..

[168] Oppliger R.A., Magnes S.A., Popowski L.A., Gisolfi C.V. (2005). Accuracy of urine specific gravity and osmolality as indicators of hydration status. Int. J. Sport Nutr. Exerc. Metab..

[169] Armstrong L.E., Soto J.A., Hacker F.T., Casa D.J., Kavouras S.A., Maresh C.M. (1998). Urinary indices during dehydration, exercise, and rehydration. Int. J. Sport Nutr..

[170] Oppliger R.A., Bartok C. (2002). Hydration testing of athletes. Sports Med..

[171] Mentes J.C., Wakefield B., Culp K. (2006). Use of a urine color chart to monitor hydration status in nursing home residents. Biol. Res. Nurs..

[172] EFSA (2008). Draft dietary reference values for water. Scientific Opinion of the Panel on Dietetic Products, Nutrition and Allergies (agreed on 11 April 2008 for release for public consultation). Eur. Food Saf. Auth..

[173] Welch B.E., Buskirk E.R., Iampietro P.F. (1958). Relation of climate and temperature to food and water intake in man. Metabolism.

[174] Collins J., Rollo I. (2014). Practical Considerations in Elite Football. Sports Sci. Exch..

[175] Ray M.L., Bryan M.W., Ruden T.M., Baier S.M., Sharp R.L., King D.S. (1998). Effect of sodium in a rehydration beverage when consumed as a fluid or meal. J. Appl. Physiol. (1985).

[176] Shirreffs S.M., Maughan R.J. (1998). Urine osmolality and conductivity as indices of hydration status in athletes in the heat. Med. Sci. Sports Exerc..

[177] Broad E.M., Burke L.M., Cox G.R., Heeley P., Riley M. (1996). Body weight changes and voluntary fluid intakes during training and competition sessions in team sports. Int. J. Sport Nutr..

[178] Maughan R.J., Leiper J.B. (1994). Fluid replacement requirements in soccer. J. Sports Sci..

[179] Shirreffs S.M., Sawka M.N. (2011). Fluid and electrolyte needs for training, competition, and recovery. J. Sports Sci..

[180] Kovacs E.M., Schmahl R.M., Senden J.M., Brouns F. (2002). Effect of high and low rates of fluid intake on post-exercise rehydration. Int. J. Sport Nutr. Exerc. Metab..

[181] Wong S.H., Williams C., Simpson M., Ogaki T. (1998). Influence of fluid intake pattern on short-term recovery from prolonged, submaximal running and subsequent exercise capacity. J. Sports Sci..

[182] Shirreffs S.M., Maughan R.J. (1998). Volume repletion after exercise-induced volume depletion in humans: Replacement of water and sodium losses. Am. J. Physiol..

[183] Nelson J.L., Robergs R.A. (2007). Exploring the potential ergogenic effects of glycerol hyperhydration. Sports Med..

[184] Freund B.J., Montain S.J., Young A.J., Sawka M.N., DeLuca J.P., Pandolf K.B., Valeri C.R. (1995). Glycerol hyperhydration: Hormonal, renal, and vascular fluid responses. J. Appl. Physiol..

[185] Latzka W.A., Sawka M.N. (2000). Hyperhydration and glycerol: Thermoregulatory effects during exercise in hot climates. Can. J. Appl. Physiol..

[186] O’Brien C., Freund B.J., Young A.J., Sawka M.N. (2005). Glycerol hyperhydration: Physiological responses during cold-air exposure. J. Appl. Physiol..

[187] (2011). IOC consensus statement on sports nutrition 2010. J. Sports Sci..

[188] Eskici G. (2016). The Importance of Vitaminsfor Soccer Players. Int. J. Vitam. Nutr. Res..

[189] Hood D.A., Kelton R., Nishio M.L. (1992). Mitochondrial adaptations to chronic muscle use: Effect of iron deficiency. Comp. Biochem. Physiol. Comp. Physiol..

[190] Reinke S., Taylor W.R., Duda G.N., von Haehling S., Reinke P., Volk H.-D., Anker S.D., Doehner W. (2012). Absolute and functional iron deficiency in professional athletes during training and recovery. Int. J. Cardiol..

[191] Escanero J.F., Villanueva J., Rojo A., Herrera A., del Diego C., Guerra M. (1997). Iron stores in professional athletes throughout the sports season. Physiol. Behav..

[192] Heisterberg M.F., Fahrenkrug J., Krustrup P., Storskov A., Kjær M., Andersen J.L. (2013). Extensive monitoring through multiple blood samples in professional soccer players. J. Strength Cond. Res..

[193] Ostojic S.M., Ahmetovic Z. (2009). Indicators of iron status in elite soccer players during the sports season. Int. J. Lab. Hematol..

[194] Villanueva J., Soria M., González-Haro C., Ezquerra L., Nieto J.L., Escanero J.F. (2011). Oral iron treatment has a positive effect on iron metabolism in elite soccer players. Biol. Trace Elem. Res..

[195] Peeling P., Dawson B., Goodman C., Landers G., Wiegerinck E.T., Swinkels D.W., Trinder D. (2009). Effects of exercise on hepcidin response and iron metabolism during recovery. Int. J. Sport Nutr. Exerc. Metab..

[196] Larson-Meyer D.E., Willis K.S. (2010). Vitamin D and athletes. Curr. Sports Med. Rep..

[197] Shuler F.D., Wingate M.K., Moore G.H., Giangarra C. (2012). Sports health benefits of vitamin d. Sports Health.

[198] Tukaj C. (2008). Adequate level of vitamin D is essential for maintaining good health. Postepy Hig. Med. Dosw..

[199] Willis K.S., Peterson N.J., Larson-Meyer D.E. (2008). Should we be concerned about the vitamin D status of athletes?. Int. J. Sport Nutr. Exerc. Metab..

[200] Hamilton B., Whiteley R., Farooq A., Chalabi H. (2014). Vitamin D concentration in 342 professional football players and association with lower limb isokinetic function. J. Sci. Med. Sport.

[201] Książek A., Zagrodna A., Dziubek W., Pietraszewski B., Ochmann B., Słowińska-Lisowska M. (2016). 25(OH)D3 Levels Relative to Muscle Strength and Maximum Oxygen Uptake in Athletes. J. Hum. Kinet..

[202] Kopeć A., Solarz K., Majda F., Słowińska-Lisowska M., Mędraś M. (2013). An evaluation of the levels of vitamin d and bone turnover markers after the summer and winter periods in polish professional soccer players. J. Hum. Kinet..

[203] Vander Slagmolen G., van Hellemondt F.J., Wielders J. (2014). Do Professional Soccer Players have a Vitamin D Status Supporting Optimal Performance in Winter time?. J. Sports Med. Doping Stud..

[204] Morton J.P., Iqbal Z., Drust B., Burgess D., Close G.L., Brukner P.D. (2012). Seasonal variation in vitamin D status in professional soccer players of the English Premier League. Appl. Physiol. Nutr. Metab..

[205] Angelini F., Marzatico F., Stesina G., Stefanini L., Bonuccelli A., Beschi S., Buonocore D., Rucci S., Tencone F. (2011). Seasonal pattern of vitamin D in male elite soccer players. J. Int. Soc. Sports Nutr..

[206] Holick M.F. (2007). Vitamin D deficiency. N. Engl. J. Med..

[207] Institute of Medicine (2011). Dietary Reference Intakes for Calcium and Vitamin D.

[208] Holick M.F., Binkley N.C., Bischoff-Ferrari H.A., Gordon C.M., Hanley D.A., Heaney R.P., Murad M.H., Weaver C.M., Endocrine S. (2011). Evaluation, treatment, and prevention of vitamin D deficiency: An Endocrine Society clinical practice guideline. J. Clin. Endocrinol. Metab..

[209] Powers S., Nelson W.B., Larson-Meyer E. (2011). Antioxidant and Vitamin D supplements for athletes: Sense or nonsense?. J. Sports Sci..

[210] Mankowski R.T., Anton S.D., Buford T.W., Leeuwenburgh C. (2015). Dietary Antioxidants as Modifiers of Physiologic Adaptations to Exercise. Med. Sci. Sports Exerc..

[211] Chun J.-M., Kwak J.-J., Choi H.-M., Park S., Lee J.-H., Kim J.-K., Jung J.W., Nho H. (2010). Antioxidant intravenous supplementation improves cardiac output during dynamic exercise in college soccer athletes. FASEB J..

[212] Ferrer M.D., Tauler P., Sureda A., Pujol P., Drobnic F., Tur J.A., Pons A. (2009). A soccer match’s ability to enhance lymphocyte capability to produce ROS and induce oxidative damage. Int. J. Sport Nutr. Exerc. Metab..

[213] Tauler P., Ferrer M.D., Sureda A., Pujol P., Drobnic F., Tur J.A., Pons A. (2008). Supplementation with an antioxidant cocktail containing coenzyme Q prevents plasma oxidative damage induced by soccer. Eur. J. Appl. Physiol..

[214] Urso M.L., Clarkson P.M. (2003). Oxidative stress, exercise, and antioxidant supplementation. Toxicology.

[215] Kelly M.K., Wicker R.J., Barstow T.J., Harms C.A. (2009). Effects of N-acetylcysteine on respiratory muscle fatigue during heavy exercise. Respir. Physiol. Neurobiol..

[216] Machefer G., Groussard C., Zouhal H., Vincent S., Youssef H., Faure H., Malardé L., Gratas-Delamarche A. (2007). Nutritional and plasmatic antioxidant vitamins status of ultra endurance athletes. J. Am. Coll. Nutr..

[217] Rankinen T., Lyytikainen S., Vanninen E., Penttila I., Rauramaa R., Uusitupa M. (1998). Nutritional status of the Finnish elite ski jumpers. Med. Sci. Sports Exerc..

[218] Palazzetti S., Rousseau A.S., Richard M.J., Favier A., Margaritis I. (2004). Antioxidant supplementation preserves antioxidant response in physical training and low antioxidant intake. Br. J. Nutr..

[219] Powers S.K., Nelson W.B., Hudson M.B. (2011). Exercise-induced oxidative stress in humans: Cause and consequences. Free Radic. Biol. Med..

[220] Teixeira V.H., Valente H.F., Casal S.I., Marques A.F., Moreira P.A. (2009). Antioxidants do not prevent postexercise peroxidation and may delay muscle recovery. Med. Sci. Sports Exerc..

[221] Coombes J.S., Powers S.K., Rowell B., Hamilton K.L., Dodd S.L., Shanely R.A., Sen C.K., Packer L. (2001). Effects of vitamin E and α-lipoic acid on skeletal muscle contractile properties. J. Appl. Physiol..

[222] McGinley C., Shafat A., Donnelly A.E. (2009). Does antioxidant vitamin supplementation protect against muscle damage?. Sports Med..

[223] Beaton L.J., Allan D.A., Tarnopolsky M.A., Tiidus P.M., Phillips S.M. (2002). Contraction-induced muscle damage is unaffected by vitamin E supplementation. Med. Sci. Sports Exerc..

[224] Theodorou A.A., Nikolaidis M.G., Paschalis V., Koutsias S., Panayiotou G., Fatouros I.G., Koutedakis Y., Jamurtas A.Z. (2011). No effect of antioxidant supplementation on muscle performance and blood redox status adaptations to eccentric training. Am. J. Clin. Nutr..

[225] Childs A., Jacobs C., Kaminski T., Halliwell B., Leeuwenburgh C. (2001). Supplementation with vitamin C and N-acetyl-cysteine increases oxidative stress in humans after an acute muscle injury induced by eccentric exercise. Free Radic. Biol. Med..

[226] Bjelakovic G., Nikolova D., Gluud L.L., Simonetti R.G., Gluud C. (2007). Mortality in randomized trials of antioxidant supplements for primary and secondary prevention: Systematic review and meta-analysis. J. Am. Med. Assoc..

[227] Braakhuis A.J., Hopkins W.G. (2015). Impact of Dietary Antioxidants on Sport Performance: A Review. Sports Med..

[228] Halliwell B. (2000). The antioxidant paradox. Lancet.

[229] Peternelj T.T., Coombes J.S. (2011). Antioxidant supplementation during exercise training: Beneficial or detrimental?. Sports Med..

[230] Tscholl P., Junge A., Dvorak J. (2008). The use of medication and nutritional supplements during FIFA World Cups 2002 and 2006. Br. J. Sports Med..

[231] Braun H., Koehler K., Geyer H., Kleinert J., Mester J., Schänzer W. (2009). Dietary supplement use among elite young German athletes. Int. J. Sport Nutr. Exerc. Metab..

[232] Dascombe B.J., Karunaratna M., Cartoon J., Fergie B., Goodman C. (2010). Nutritional supplementation habits and perceptions of elite athletes within a state-based sporting institute. J. Sci. Med. Sport.

[233] Maughan R.J., Greenhaff P.L., Hespel P. (2011). Dietary supplements for athletes: Emerging trends and recurring themes. J. Sports Sci..

[234] Read M.M., Cisar C. (2001). The Influence of Varied Rest Interval Lengths on Depth Jump Performance. J. Strength Cond. Res..

[235] European Food Safety Authority (2013). Food Supplements. http://www.efsa.europa.eu/en/topics/topic/supplements.htm.

[236] Maughan R.J. (2013). Quality assurance issues in the use of dietary supplements, with special reference to protein supplements. J. Nutr..

[237] Geyer H., Parr M.K., Koehler K., Mareck U., Schänzer W., Thevis M. (2008). Nutritional supplements cross-contaminated and faked with doping substances. J. Mass Spectrom..

[238] Judkins C., Prock P. (2013). Supplements and inadvertent doping—How big is the risk to athletes?. Med. Sport Sci..

[239] Geyer H., Parr M.K., Mareck U., Reinhart U., Schrader Y., Schänzer W. (2004). Analysis of Non-Hormonal Nutritional Supplements for Anabolic-Androgenic Steroids—Results of an International Study. Int. J. Sports Med..

[240] Burke L.M., Castell L.M., Stear S.J. (2009). A-Z of supplements: Dietary supplements, sports nutrition foods and ergogenic aids for health and performance—Part 1. Br. J. Sports Med..

[241] Geyer H., Braun H., Burke L.M., Stear S.J., Castell L.M. (2011). A-Z of nutritional supplements: Dietary supplements, sports nutrition foods and ergogenic aids for health and performance—Part 22. Br. J. Sports Med..

[242] Hoffman J.R., Emerson N.S., Stout J.R. (2012). beta-Alanine supplementation. Curr. Sports Med. Rep..

[243] Quesnele J.J., Laframboise M.A., Wong J.J., Kim P., Wells G.D. (2014). The effects of beta-alanine supplementation on performance: A systematic review of the literature. Int. J. Sport Nutr. Exerc. Metab..

[244] Trexler E.T., Smith-Ryan A.E., Stout J.R., Hoffman J.R., Wilborn C.D., Sale C., Kreider R.B., Jager R., Earnest C.P., Bannock L. (2015). International society of sports nutrition position stand: Beta-Alanine. J. Int. Soc. Sports Nutr..

[245] Goldstein E.R., Ziegenfuss T., Kalman D., Kreider R., Campbell B., Wilborn C., Taylor L., Willoughby D., Stout J., Graves B.S. (2010). International society of sports nutrition position stand: Caffeine and performance. J. Int. Soc. Sports Nutr..

[246] Ali A., O’Donnell J., Von Hurst P., Foskett A., Holland S., Starck C., Rutherfurd-Markwick K. (2016). Caffeine ingestion enhances perceptual responses during intermittent exercise in female team-game players. J. Sports Sci..

[247] Smith R.N., Agharkar A.S., Gonzales E.B. (2014). A review of creatine supplementation in age-related diseases: More than a supplement for athletes. F1000Research.

[248] Lanhers C., Pereira B., Naughton G., Trousselard M., Lesage F.X., Dutheil F. (2015). Creatine Supplementation and Lower Limb Strength Performance: A Systematic Review and Meta-Analyses. Sports Med..

[249] Andres S., Ziegenhagen R., Trefflich I., Pevny S., Schultrich K., Braun H., Schanzer W., Hirsch-Ernst K.I., Schafer B., Lampen A. (2016). Creatine and creatine forms intended for sports nutrition. Mol. Nutr. Food Res..

[250] Wylie L.J., Mohr M., Krustrup P., Jackman S.R., Ermiotadis G., Kelly J., Black M.I., Bailey S.J., Vanhatalo A., Jones A.M. (2013). Dietary nitrate supplementation improves team sport-specific intense intermittent exercise performance. Eur. J. Appl. Physiol..

[251] Thompson C., Vanhatalo A., Jell H., Fulford J., Carter J., Nyman L., Bailey S.J., Jones A.M. (2016). Dietary nitrate supplementation improves sprint and high-intensity intermittent running performance. Nitric Oxide.

[252] Bishop D. (2010). Dietary supplements and team-sport performance. Sports Med..

[253] Bellinger P.M. (2014). beta-Alanine supplementation for athletic performance: An update. J. Strength Cond. Res..

[254] Blancquaert L., Everaert I., Derave W. (2015). Beta-alanine supplementation, muscle carnosine and exercise performance. Curr. Opin. Clin. Nutr. Metab. Care.

[255] Begum G., Cunliffe A., Leveritt M. (2005). Physiological role of carnosine in contracting muscle. Int. J. Sport Nutr. Exerc. Metab..

[256] Hill C.A., Harris R.C., Kim H.J., Harris B.D., Sale C., Boobis L.H., Kim C.K., Wise J.A. (2007). Influence of beta-alanine supplementation on skeletal muscle carnosine concentrations and high intensity cycling capacity. Amino Acids.

[257] Hoffman J.R., Ratamess N.A., Faigenbaum A.D., Ross R., Kang J., Stout J.R., Wise J.A. (2008). Short-duration beta-alanine supplementation increases training volume and reduces subjective feelings of fatigue in college football players. Nutr. Res..

[258] Hoffman J., Ratamess N., Kang J., Mangine G., Faigenbaum A., Stout J. (2006). Effect of creatine and beta-alanine supplementation on performance and endocrine responses in strength/power athletes. Int. J. Sport Nutr. Exerc. Metab..

[259] Saunders B., Sunderland C., Harris R.C., Sale C. (2012). β-alanine supplementation improves YoYo intermittent recovery test performance. J. Int. Society Sports Nutr..

[260] Van Thienen R., Van Proeyen K., Vanden Eynde B., Puype J., Lefere T., Hespel P. (2009). Beta-alanine improves sprint performance in endurance cycling. Med. Sci. Sports Exerc..

[261] Tarnopolsky M.A. (2010). Caffeine and Creatine Use in Sport. Ann. Nutr. Metab..

[262] Foskett A., Ali A., Gant N. (2009). Caffeine enhances cognitive function and skill performance during simulated soccer activity. Int. J. Sport Nutr. Exerc. Metab..

[263] Gant N., Ali A., Foskett A. (2010). The influence of caffeine and carbohydrate coingestion on simulated soccer performance. Int. J. Sport Nutr. Exerc. Metab..

[264] Lara B., Gonzalez-Millan C., Salinero J.J., Abian-Vicen J., Areces F., Barbero-Alvarez J.C., Munoz V., Portillo L.J., Gonzalez-Rave J.M., Del Coso J. (2014). Caffeine-containing energy drink improves physical performance in female soccer players. Amino Acids.

[265] Andrade-Souza V.A., Bertuzzi R., de Araujo G.G., Bishop D., Lima-Silva A.E. (2015). Effects of isolated or combined carbohydrate and caffeine supplementation between 2 daily training sessions on soccer performance. Appl. Physiol. Nutr. Metab..

[266] Pettersen S.A., Krustrup P., Bendiksen M., Randers M.B., Brito J., Bangsbo J., Jin Y., Mohr M. (2014). Caffeine supplementation does not affect match activities and fatigue resistance during match play in young football players. J. Sports Sci..

[267] Wallimann T., Wyss M., Brdiczka D., Nicolay K., Eppenberger H.M. (1992). Intracellular compartmentation, structure and function of creatine kinase isoenzymes in tissues with high and fluctuating energy demands: The ‘phosphocreatine circuit’ for cellular energy homeostasis. Biochem. J..

[268] Cheetham M.E., Boobis L.H., Brooks S., Williams C. (1986). Human muscle metabolism during sprint running. J. Appl. Physiol. (1985).

[269] Rae C., Digney A.L., McEwan S.R., Bates T.C. (2003). Oral creatine monohydrate supplementation improves brain performance: A double-blind, placebo-controlled, cross-over trial. Proc. R. Soc. B Biol. Sci..

[270] Ostojic S.M. (2004). Creatine supplementation in young soccer players. Int. J. Sport Nutr. Exerc. Metab..

[271] Mujika I., Padilla S., Ibanez J., Izquierdo M., Gorostiaga E. (2000). Creatine supplementation and sprint performance in soccer players. Med. Sci. Sports Exerc..

[272] Cox G., Mujika I., Tumilty D., Burke L. (2002). Acute creatine supplementation and performance during a field test simulating match play in elite female soccer players. Int. J. Sport Nutr. Exerc. Metab..

[273] Larson-Meyer D.E., Hunter G.R., Trowbridge C.A., Turk J.C., Ernest J.M., Torman S.L., Harbin P.A. (2000). The Effect of Creatine Supplementation on Muscle Strength and Body Composition During Off-Season Training in Female Soccer Players. J. Strength Cond. Res..

[274] Claudino J.G., Mezencio B., Amaral S., Zanetti V., Benatti F., Roschel H., Gualano B., Amadio A.C., Serrao J.C. (2014). Creatine monohydrate supplementation on lower-limb muscle power in Brazilian elite soccer players. J. Int. Soc. Sports Nutr..

[275] Roberts P.A., Fox J., Peirce N., Jones S.W., Casey A., Greenhaff P.L. (2016). Creatine ingestion augments dietary carbohydrate mediated muscle glycogen supercompensation during the initial 24 h of recovery following prolonged exhaustive exercise in humans. Amino Acids.

[276] Williams J., Abt G., Kilding A.E. (2014). Effects of Creatine Monohydrate Supplementation on Simulated Soccer Performance. Int. J. Sports Physiol. Perform..

[277] Wilder N., Deivert R.G., Hagerman F., Gilders R. (2001). The Effects of Low-Dose Creatine Supplementation Versus Creatine Loading in Collegiate Football Players. J. Athl. Train..

[278] Syrotuik D.G., Bell G.J. (2004). Acute creatine monohydrate supplementation: A descriptive physiological profile of responders vs. nonresponders. J. Strength Cond. Res..

[279] Antonio J., Ciccone V. (2013). The effects of pre versus post workout supplementation of creatine monohydrate on body composition and strength. J. Int. Soc. Sports Nutr..

[280] Candow D.G., Vogt E., Johannsmeyer S., Forbes S.C., Farthing J.P. (2015). Strategic creatine supplementation and resistance training in healthy older adults. Appl. Physiol. Nutr. Metab..

[281] Candow D.G., Chilibeck P.D., Burke D.G., Mueller K.D., Lewis J.D. (2011). Effect of different frequencies of creatine supplementation on muscle size and strength in young adults. J. Strength Cond. Res..

[282] Green A.L., Hultman E., Macdonald I.A., Sewell D.A., Greenhaff P.L. (1996). Carbohydrate ingestion augments skeletal muscle creatine accumulation during creatine supplementation in humans. Am. J. Physiol..

[283] Steenge G.R., Simpson E.J., Greenhaff P.L. (2000). Protein- and carbohydrate-induced augmentation of whole body creatine retention in humans. J. Appl. Physiol. (1985).

[284] Schilling B.K., Stone M.H., Utter A., Kearney J.T., Johnson M., Coglianese R., Smith L., O’Bryant H.S., Fry A.C., Starks M. (2001). Creatine supplementation and health variables: A retrospective study. Med. Sci. Sports Exerc..

[285] Larsen F.J., Weitzberg E., Lundberg J.O., Ekblom B. (2007). Effects of dietary nitrate on oxygen cost during exercise. Acta Physiol. (Oxf. Engl.).

[286] Jones A.M. (2014). Dietary nitrate supplementation and exercise performance. Sports Med..

[287] Jonvik K.L., Nyakayiru J., Van Dijk J.W., Wardenaar F.C., Van Loon L.J., Verdijk L.B. (2017). Habitual Dietary Nitrate Intake in Highly Trained Athletes. Int. J. Sport Nutr. Exerc. Metab..

[288] Thompson C., Wylie L.J., Fulford J., Kelly J., Black M.I., McDonagh S.T., Jeukendrup A.E., Vanhatalo A., Jones A.M. (2015). Dietary nitrate improves sprint performance and cognitive function during prolonged intermittent exercise. Eur. J. Appl. Physiol..

[289] Saunders B., Sale C., Harris R.C., Sunderland C. (2014). Effect of sodium bicarbonate and Beta-alanine on repeated sprints during intermittent exercise performed in hypoxia. Int. J. Sport Nutr. Exerc. Metab..

[290] Cameron S.L., McLay-Cooke R.T., Brown R.C., Gray A.R., Fairbairn K.A. (2010). Increased blood pH but not performance with sodium bicarbonate supplementation in elite rugby union players. Int. J. Sport Nutr. Exerc. Metab..

[291] Krustrup P., Ermidis G., Mohr M. (2015). Sodium bicarbonate intake improves high-intensity intermittent exercise performance in trained young men. J. Int. Soc. Sports Nutr..

